# Silencing of Epidermal Growth Factor-like Domain 8 Promotes Proliferation and Cancer Aggressiveness in Human Ovarian Cancer Cells by Activating ERK/MAPK Signaling Cascades

**DOI:** 10.3390/ijms26010274

**Published:** 2024-12-31

**Authors:** Yong-Jung Song, Ji-Eun Kim, Lata Rajbongshi, Ye-Seon Lim, Ye-Jin Ok, Seon-Yeong Hwang, Hye-Yun Park, Jin-Eui Lee, Sae-Ock Oh, Byoung-Soo Kim, Dongjun Lee, Hwi-Gon Kim, Sik Yoon

**Affiliations:** 1 Department of Obstetrics and Gynecology, Pusan National University Yangsan Hospital and Pusan National University College of Medicine, Yangsan 50612, Republic of Korea; gynsong@gmail.com (Y.-J.S.); bislsan@naver.com (H.-G.K.); 2 Research Institute for Convergence of Biomedical Science and Technology, Pusan National University Yangsan Hospital, Yangsan 50612, Republic of Korea; 3 Department of Anatomy and Convergence Medical Sciences, Pusan National University College of Medicine, Yangsan 50612, Republic of Korea; ji.eun@pusan.ac.kr (J.-E.K.); latapharm@gmail.com (L.R.); yeseonlim@pusan.ac.kr (Y.-S.L.); yj0429@pusan.ac.kr (Y.-J.O.); anatomy2017@pusan.ac.kr (S.-Y.H.); qkrvlfg123@naver.com (H.-Y.P.); wlsdbs88@naver.com (J.-E.L.);; 4 School of Biomedical Convergence Engineering, Pusan National University, Yangsan 50612, Republic of Korea; bskim7@pusan.ac.kr; 5 Department of Convergence Medicine, Pusan National University College of Medicine, Yangsan 50612, Republic of Korea; lee.dongjun@pusan.ac.kr

**Keywords:** epidermal growth factor-like domain 8 (EGFL8), ovarian cancer, EMT, cancer aggressiveness, chemoresistance, ERK/MAPK

## Abstract

Ovarian cancer (OC) is the second most common female reproductive cancer and the most lethal gynecological malignancy worldwide. Most human OCs are characterized by high rates of drug resistance and metastasis, leading to poor prognosis. Improving the outcomes of patients with relapsed and treatment-resistant OC remains a challenge. This study aimed to investigate the role of epidermal growth factor-like domain 8 (EGFL8) in human OC by examining the effects of siRNA-mediated EGFL8 knockdown on cancer progression. EGFL8 knockdown in human OC cells promoted aggressive traits associated with cancer progression, including enhanced proliferation, colony formation, migration, invasion, chemoresistance, and reduced apoptosis. Additionally, knockdown upregulated the expression of epithelial–mesenchymal transition (EMT) markers (Snail, Twist1, Zeb1, Zeb2, and vimentin) and cancer stem cell biomarkers (Oct4, Sox2, Nanog, KLF4, and ALDH1A1), and increased the expression of matrix metallopeptidases (MMP-2 and MMP-9), drug resistance genes (MDR1 and MRP1), and Notch1. Low EGFL8 expression also correlated with poor prognosis in human OC. Overall, this study provides crucial evidence that EGFL8 inhibits the proliferation and cancer aggressiveness of human OC cells by suppressing ERK/MAPK signaling. Therefore, EGFL8 may serve as a valuable prognostic biomarker and a potential target for developing novel human OC therapies.

## 1. Introduction

Ovarian cancer (OC), the most lethal gynecological malignancy, is the seventh most prevalent cancer among women and the second most common gynecological cancer worldwide [1]. Most human OCs are characterized by an acute onset, high rates of distant metastasis, extensive invasion, and drug resistance, resulting in a poor prognosis [2]. Despite considerable progress in diagnostics and therapies, the five-year survival rate for advanced-stage human OC remains below 30%, primarily because of the lack of obvious early symptoms, the absence of effective early detection methods, and late-stage diagnosis [3].

The standard initial treatment for human OC is primary cytoreductive surgery, followed by platinum- and taxane-based chemotherapy. Although the response rate to initial treatment is high, tumor recurrence is a common issue in patients undergoing OC treatment. Approximately 70% of patients with OC experience relapse after primary cytoreductive surgery and standard first-line chemotherapy [4,5]. In this context, it is important to emphasize that poor prognosis and high recurrence rates are associated with the development of chemoresistance, a malignant phenotype that significantly limits the efficacy of chemotherapy after long-term treatment with first-line drugs such as paclitaxel and cisplatin [6,7,8,9]. Overcoming chemoresistance remains a major challenge for OC research. Target identification is a critical step in the development of potential therapeutic drugs. Thus, understanding the molecular mechanisms that profoundly influence cancer progression, including tumor cell growth, survival, migration, invasion, metastasis, and acquisition of resistance, is crucial for developing targeted treatments for human OC.

The epidermal growth factor (EGF)-like domain (EGFL), an evolutionarily conserved region found in many vertebrate proteins, consists of 30–40 amino acids and shares significant homology with EGF [10,11,12]. EGFL proteins, which have one or more EGFL repeats, are generally involved in protein–protein interactions and play essential roles in key biological processes such as proliferation, differentiation, apoptosis, adhesion, migration, angiogenesis, and tumor development by binding to receptors [13,14,15,16]. To date, several members of the EGFL family were identified, including EGFL2, EGFL3, EGFL5, EGFL6, EGFL7, EGFL8, and EGFL9. EGFL2, EGFL5, and EGFL9 are integral membrane proteins with transmembrane domains, whereas EGFL3, EGFL6, EGFL7, and EGFL8 are secreted proteins that lack transmembrane domains [14,17].

EGFL8, originally identified as a paralog of EGFL7, is a newly discovered member of the EGFL family. As a highly evolutionarily conserved protein domain, EGFL8 is involved in several cellular processes, including blood coagulation, fibrinolysis, cell adhesion, and neural and vertebrae development [10]. Although few studies have focused on the characterization and biological function of EGFL8 in normal cells and tissues, its expression was detected in diverse mouse tissue types, including the ovaries, uterus, testis, epididymis, ileum, colon, and stomach [18]. Additionally, an EGFL8-mediated inhibitory effect was observed in mouse thymic epithelial cells and thymocytes [19,20].

Notably, EGFL8 expression is downregulated in gastric and colorectal cancers, and this downregulation is significantly correlated with peritoneal dissemination and high tumor–node–metastasis (TNM) stage, highlighting its potential role in cancer development [21,22]. Moreover, a recent study revealed that EGFL8 expression was significantly decreased in hepatocellular carcinoma tissues and cell lines, and its downregulation was linked to more aggressive disease features, including multiple nodules, vascular invasion, advanced TNM stage, lung metastasis, poor prognosis, and decreased cell apoptosis and migration via the activation of the Notch signaling pathway [23]. Furthermore, EGFL8 exerts an antiproliferative effect on neuroblastoma cells, and higher EGFL8 expression in peripheral neuroblastic tumors correlates with improved clinical outcomes and significantly higher 5-year overall survival rates, potentially owing to its neuritogenic effect [24].

However, a comprehensive analysis of the biological characteristics and functional significance of EGFL8 in tumors is lacking. In this study, we investigated the role of EGFL8 in cell proliferation, spheroid formation, clonogenesis, apoptosis, migration, invasion, and malignant progression, including the induction of chemoresistance, epithelial–mesenchymal transition (EMT), stemness, and cancer aggravation in human OC cells. This is the first study to report that EGFL8 is essential for human OC malignancy and progression, providing important evidence for its potential role in regulating human OC progression and contributing to the development of new and effective targeted therapies for human OC.

## 2. Results

### 2.1. EGFL8 Expression Is Downregulated in Human OC Tissues

**Figure 1:**
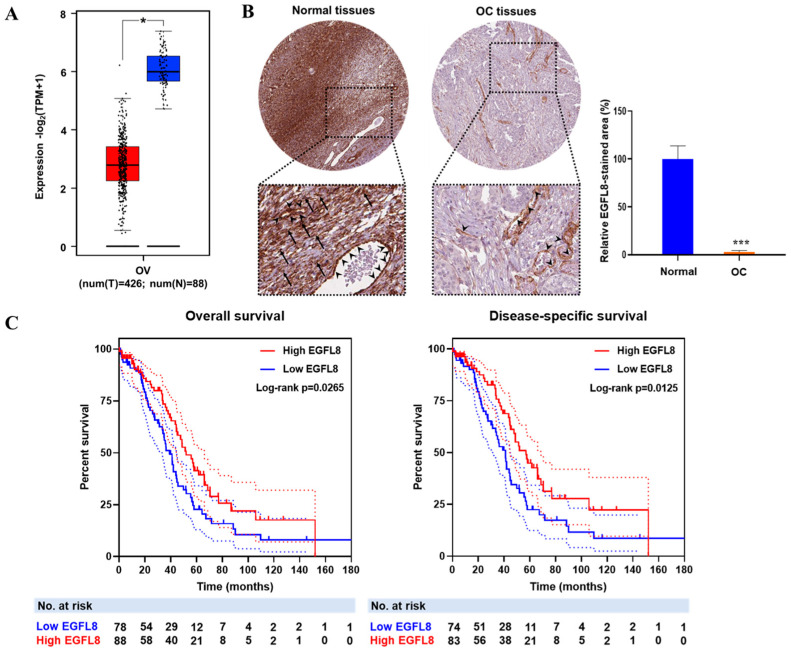
EGFL8 expression and its relationship with prognosis of patients with ovarian cancer (OC). (**A**) Relative expression levels of EGFL8 mRNA in human OC tissues (*n* = 426, red box) and normal ovarian tissues (*n* = 88, blue box) analyzed using GEPIA2 platform based on TCGA and GTEx data. (**B**) Representative images show EGFL8 protein expression in ovarian stromal cells (arrows) and vascular endothelial cells (arrowheads) of human OC and normal ovarian tissues, obtained from Human Protein Atlas database, as determined by immunohistochemical staining (magnification, 40×). Bar graphs represent area of immunostained regions in each group. (**C**) Overall survival (OS) and disease-specific survival (DSS) analysis of EGFL8 expression levels in patients with OC. Number of patients at risk (No. at risk) at different time points is displayed on graph. Significant differences between normal and OC groups are indicated by * *p* < 0.05 and *** *p* < 0.001. TCGA, The Cancer Genome Atlas; UALCAN, University of Alabama at Birmingham Cancer.

To investigate the clinical relevance of EGFL8, the correlation between EGFL8 mRNA expression and OC patient data from TCGA and GTEx databases was analyzed using the GEPIA2 web portal (Figure 1A). EGFL8 protein expression, determined by immunohistochemical staining, was obtained from the Human Protein Atlas and analyzed using ImageJ software (Figure 1B). The analysis revealed that EGFL8 mRNA was highly expressed in normal human ovarian tissues, particularly in ovarian stromal cells, as well as in vascular endothelial and smooth muscle cells, whereas its expression was significantly reduced in human OC tissues (Figure 1A,B). Notably, a drastic suppression of EGFL8 protein was observed in ovarian stromal cells, in contrast to vascular endothelial and smooth muscle cells, which showed minimal changes (Figure 1B).

Furthermore, the survival analysis revealed that lower EGFL8 expression was associated with worse overall survival OS (*p* = 0.0265) and disease-specific survival (DSS; *p* = 0.0125) in patients with OC (Figure 1C). Taken together, these findings suggest that EGFL8 expression is altered during cancer development and has a prognostic value in human OC.

### 2.2. EGFL8 Is Expressed in Human OC Cells

**Figure 2:**
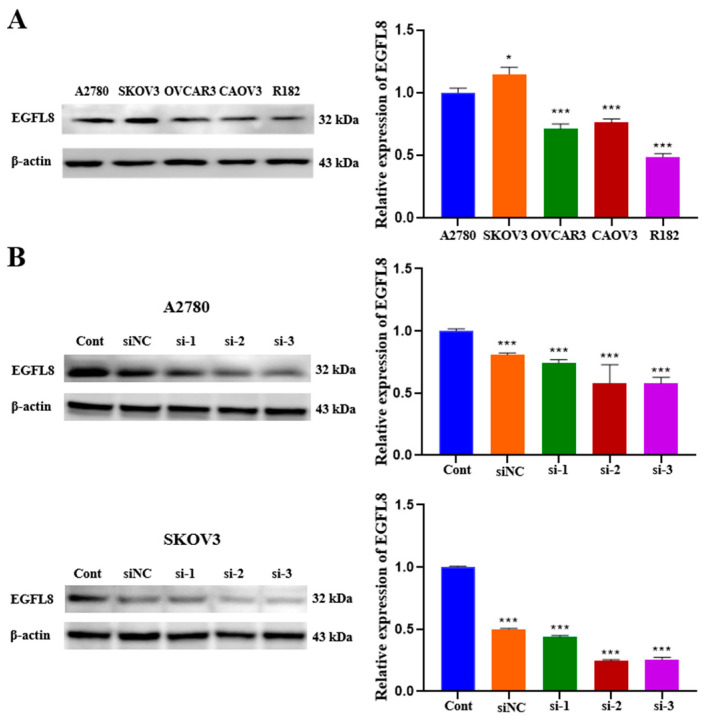
EGFL8 protein expression in human OC cells as detected by Western blot analysis. (**A**) EGFL8 protein expression in various human OC cell lines. (**B**) Western blot analysis for EGFL8 siRNA transfection efficiency, comparing non-transfected control (Cont), scrambled negative control siRNA (siNC), and EGFL8 siRNA-transfected A2780 and SKOV3 cells. Data represent mean ± standard deviation (SD) of three independent experiments. * *p* < 0.05 and *** *p* < 0.001 vs. respective control.

We examined EGFL8 expression in a series of human OC cell lines. As is consistent with the data obtained from OC clinical specimens, EGFL8 protein expression was confirmed in various human OC cell lines, including A2780, SKOV3, OVCAR3, CAOV3, and R182, by Western blot analysis (Figure 2A). Although EGFL8 expression was detectable in all five cell lines, A2780 and SKOV3 cells exhibited higher levels than the other cell lines (Figure 2A). Therefore, A2780 and SKOV3 cells were selected for further experiments based on this observation.

To investigate the role of EGFL8 in human OC cells, we knocked down EGFL8 in A2780 and SKOV3 cells by transfection with three distinct EGFL8-specific siRNAs (designated as siRNA-1, siRNA-2, and siRNA-3). The downregulation of EGFL8 expression was confirmed by comparing EGFL8 protein levels in cells transfected with scrambled negative control siRNA (siNC) and three EGFL8-specific siRNAs (Figure 2B). Notably, EGFL8 protein expression in the siRNA-transfected groups was significantly reduced compared to that in the scrambled control group at 24 h post-transfection. In A2780 cells, transfection with siRNA-1, siRNA-2, and siRNA-3 resulted in a 0.8-fold (*p* < 0.001), 0.6-fold (*p* < 0.001), and 0.6-fold (*p* < 0.001) decrease in EGFL8 expression, respectively, compared to the scrambled control group; in SKOV3 cells, decreases were 0.4-fold (*p* < 0.001), 0.2-fold (*p* < 0.001), and 0.3-fold (*p* < 0.001), respectively (Figure 2B). Consequently, we selected siRNA-2 and siRNA-3, henceforth designated as siEGFL8#1 and siEGFL8#2, respectively, for EGFL8 mRNA silencing in this study.

### 2.3. Knockdown of EGFL8 Accelerates Proliferation of Human OC Cells

**Figure 3:**
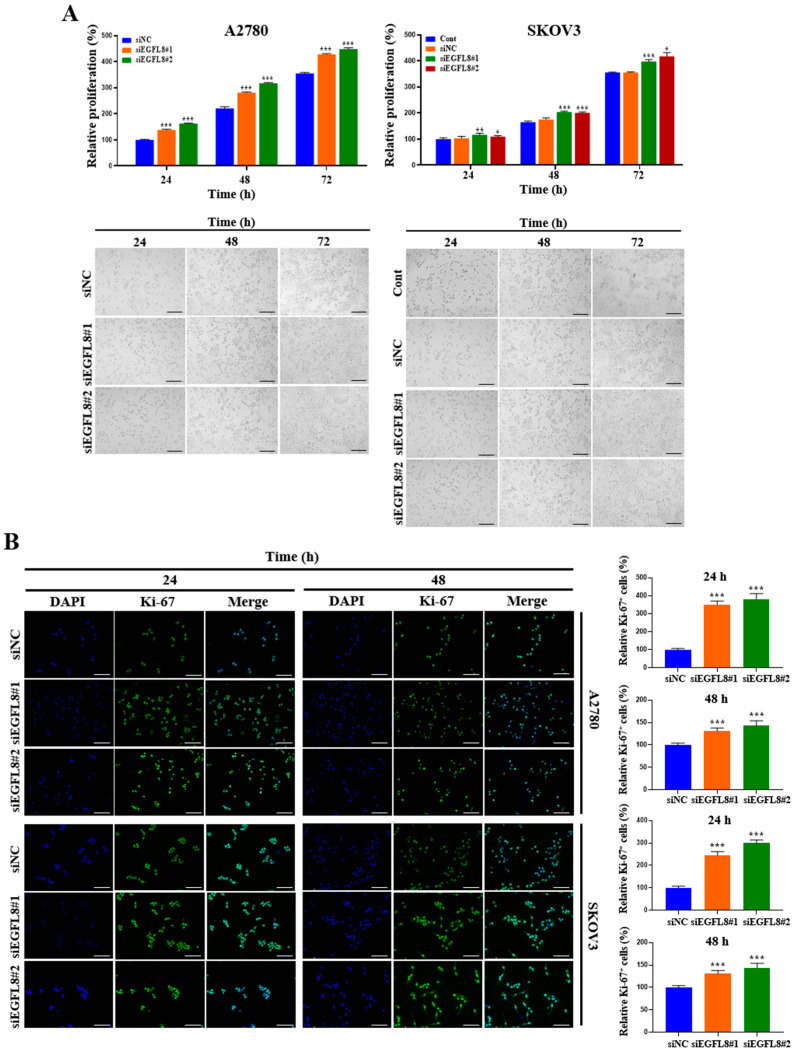
Effects of EGFL8 knockdown on cell proliferation. (**A**) Bar graphs showing proliferation of non-transfected control (control), scrambled negative control siEGFL8 (siNC), and siEGFL8-transfected A2780 and SKOV3 cells on days 1, 2, and 3, as assessed by WST-1 assay. Representative phase-contrast microscopic images depict cell morphology in siNC and siEGFL8-transfected A2780 and SKOV3 cells on days 1, 2, and 3. Scale bars = 150 μm. (**B**) Representative fluorescence micrographs of Ki-67 staining (blue: DAPI; green: Ki-67) in siNC and siEGFL8-transfected A2780 and SKOV3 cells at 24 and 48 h. Relative percentage of Ki-67^+^ cells was calculated by dividing number of cells stained with Ki-67 by total number of cells stained with DAPI. Scale bars = 150 μm. All data are expressed as relative values compared to scrambled control group. Data represent mean ± SD of three independent experiments. * *p* < 0.05, ** *p* < 0.01, and *** *p* < 0.001 vs. respective scrambled control group.

A water-soluble tetrazolium (WST)-1-based colorimetric cell proliferation assay was used to explore the effect of silencing EGFL8 on cell proliferation. At 24, 48, and 72 h, proliferation rates in the siEGFL8#1 group increased by 1.4-fold (*p* < 0.001), 1.3-fold (*p* < 0.001), and 1.2-fold (*p* < 0.001), respectively, for A2780 cells and by 1.1-fold (*p* < 0.001), 1.2-fold (*p* < 0.001), and 1.1-fold (*p* < 0.001) for SKOV3 cells, respectively, compared to the scrambled control group (Figure 3A). In the siEGFL8#2 group, the corresponding increases at 24, 48, and 72 h were 1.6-fold (*p* < 0.001), 1.4-fold (*p* < 0.001), and 1.3-fold (*p* < 0.001) for A2780 cells and 1.1-fold (*p* < 0.001), 1.2-fold (*p* < 0.001), and 1.2-fold (*p* < 0.001) for SKOV3 cells, respectively, compared to the scrambled control group (Figure 3A).

Furthermore, these results were consistent with the morphological characteristics observed using phase-contrast microscopy (Figure 3A) and immunocytochemical staining for the cell cycle-associated nuclear antigen Ki-67, a cellular marker for proliferation (Figure 3B). At 24 and 48 h in the siEGFL8#1 group, the number of Ki-67^+^ cells increased significantly by 3.5-fold (*p* < 0.001) and 2.0-fold (*p* < 0.001) for A2780 cells, and by 2.4-fold (*p* < 0.001) and 1.3-fold (*p* < 0.001) for SKOV3 cells, respectively, compared to the scrambled control group (Figure 3B). Similarly, in the siEGFL8#2 group at 24 and 48 h, the number of Ki-67^+^ cells increased by 3.8-fold (*p* < 0.001) and 2.1-fold (*p* < 0.001) for A2780 cells, and by 3.0-fold (*p* < 0.001) and 1.4-fold (*p* < 0.001) for SKOV3 cells, respectively, compared to the scrambled control group (Figure 3B). These data suggest that EGFL8 is an essential regulator of human OC cell proliferation.

### 2.4. Knockdown of EGFL8 Enhances Clonogenecity and Spheroid Growth of Human OC Cells

**Figure 4:**
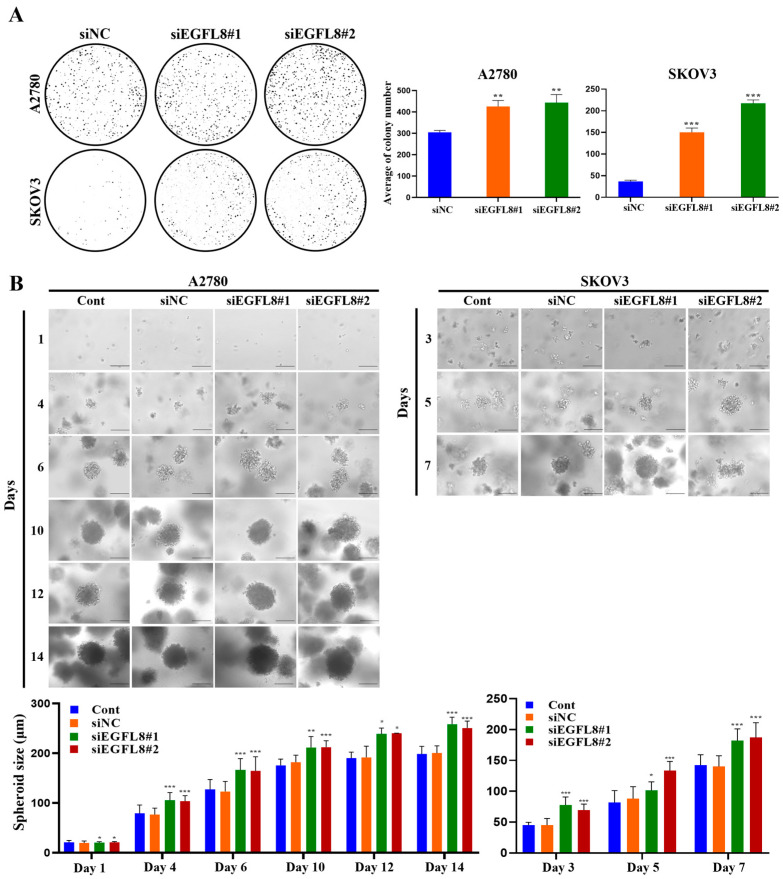
Effects of EGFL8 knockdown on colony formation and spheroid growth of human OC cells. (**A**) Colony formation assay showing siEGFL8 transfection stimulated colony formation in scrambled negative control siEGFL8 (siNC), and siEGFL8-transfected A2780 and SKOV3 cells on day 7 (magnification, 40×). (**B**) Representative phase-contrast microscopic images of OC cell spheroids generated by 3D cell culture in siNC and siEGFL8-transfected A2780 cells on days 1, 4, 6, 10, 12 and 14, and SKOV3 cells on days 3, 5, and 7. Scale bars = 150 μm. All data are expressed as relative values compared to scrambled control groups. Data represent mean ± SD of three independent experiments. * *p* < 0.05, ** *p* < 0.05, and *** *p* < 0.001 vs. respective scrambled control group.

The clonogenicity assay revealed that the colony-forming ability of the siEGFL8#1 group was significantly greater than that of the scrambled control group on day 7, with a 1.4-fold (*p* < 0.01) and 4.1-fold (*p* < 0.001) increase in A2780 and SKOV3 cells, respectively (Figure 4A). In the siEGFL8#2 group, the corresponding increases on day 7 were 1.5-fold (*p* < 0.01) and 5.9-fold (*p* < 0.001), respectively (Figure 4A).

Figure 4B shows the phase-contrast microscopy images of 3D human OC cells over time. A2780 and SKOV3 cells began to form multiple spheroids by day 3, and the number of spheroids gradually increased over time (Figure 4B). The average diameters of spheroids in the non-transfected control measured were 79.5, 127.0, 175.5, 190.1, and 198.3 µm on days 4, 6, 10, 12, and 14, respectively, for A2780 cells; and 45.3, 81.7, and 142.5 µm on days 3, 5, and 7, respectively, for SKOV3 cells (Figure 4B). The average diameters of spheroids in the scrambled control group measured were 76.6, 123.1, 181.8, 191.6, and 200.2 µm on days 4, 6, 10, 12, and 14, respectively, for A2780 cells; and 45.2, 87.9, and 140.4 µm on days 3, 5, and 7, respectively, for SKOV3 cells (Figure 4B). Spheroids derived from both A2780 and SKOV3 cells were similar in size over time, although the growth rate differed slightly between the cell types (SKOV3 > A2780; Figure 4B). After day 6, nearly all spheroids from both cell types measured over 120 µm in diameter, suggesting that MC-B hydrogels provide a supportive environment for human OC cell spheroid growth.

Morphometric analysis by phase-contrast light microscopy revealed that spheroid formation in the siEGFL8#1 group was significantly suppressed compared to the scrambled control group on days 4, 6, 10, 12, and 14 for A2780 cells, with 1.4-fold (*p* < 0.01), 1.4-fold (*p* < 0.01), 1.2-fold (*p* < 0.05), 1.2-fold (*p* < 0.01), and 1.3-fold (*p* < 0.001) decreases, respectively; and on days 3, 5, and 7 for SKOV3 cells, with 1.7-fold (*p* < 0.01), 1.2-fold (*p* < 0.01), and 1.3-fold (*p* < 0.01) decreases, respectively (Figure 4B). In the siEGFL8#2 group, the corresponding decreases on days 4, 6, 10, 12, and 14 for A2780 cells were 1.4-fold (*p* < 0.01), 1.3-fold (*p* < 0.05), 1.2-fold (*p* < 0.05), 1.3-fold (*p* < 0.05), and 1.3-fold (*p* < 0.001), respectively; and on days 3, 5, and 7 for SKOV3 cells, 1.5-fold (*p* < 0.01), 1.5-fold (*p* < 0.001), and 1.3-fold (*p* < 0.01), respectively (Figure 4B). These results suggested that EGFL8 is an essential regulator of human OC cell colony formation and spheroid growth.

### 2.5. Knockdown of EGFL8 Promotes the Metastatic Potentials of Human OC Cells

**Figure 5:**
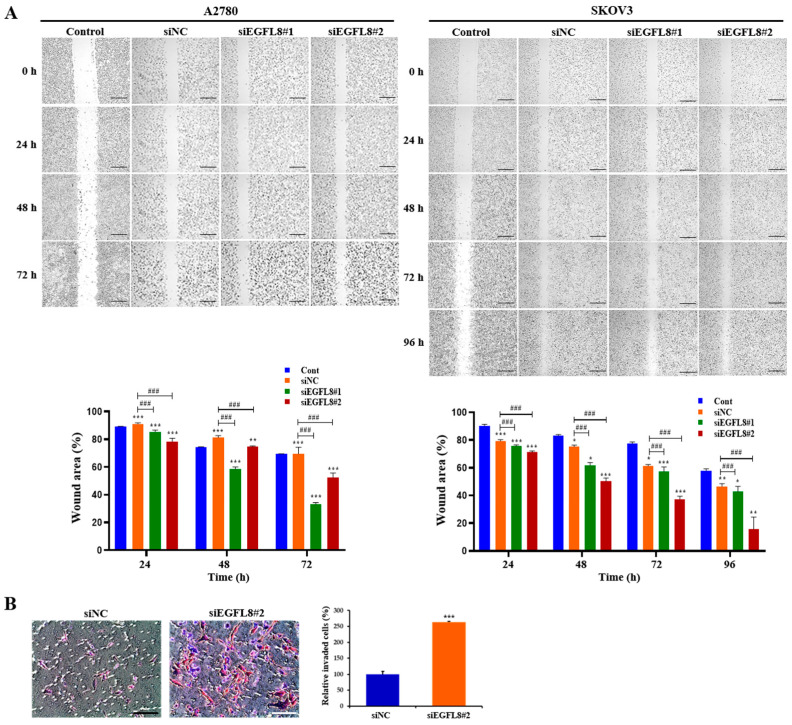
Effects of EGFL8 silencing on the migration and invasion of A2780 and SKOV3 cells. (**A**) Representative phase-contrast microscopy images from wound healing assay showing transfected and non-transfected A2780 and SKOV3 cells migrating into cell-free space. EGFL8 siRNA-transfected cells have significantly increased migratory ability compared with non-transfected control (Cont) and scrambled negative control siRNA (siNC) groups. Scale bars = 650 μm. (**B**) Representative images of transwell invasion assay with A2780 cells. Data represent mean ± SD of three independent experiments. Scale bars = 75 μm. * *p* < 0.05, ** *p* < 0.01, and *** *p* < 0.001 vs. respective non-transfected control group. ^###^ *p* < 0.001 vs. respective scrambled control group.

To further explore the effects of EGFL8 on the metastatic potential of human OC cells, wound healing and transwell invasion assays were conducted on A2780 and SKOV3 cells to evaluate their migratory and invasive abilities. Images of the scratch areas in the wound healing assay at 0, 24, 48, 72, and 96 h are shown in Figure 5. For A2780 cells in the scrambled control group, the wound closure rates at 24, 48, and 72 h were 96.8%, 92.8%, and 92.1%, respectively (Figure 5A). In the siEGFL8#1 group, these rates were 88.2%, 79.4%, and 60.4%; in the siEGFL8#2 group, they were 84.6%, 76.8%, and 56.5% (Figure 5A). This demonstrates that the migration ability of the siEGFL8#1 group was significantly suppressed at 24, 48, and 72 h compared to that of the scrambled control group, with decreases of 0.9-fold (*p* < 0.001), 0.9-fold (*p* < 0.001), and 0.7-fold (*p* < 0.001), respectively. In the siEGFL8#2 group, cell migration was reduced by 0.9-fold (*p* < 0.001), 0.8-fold (*p* < 0.001), and 0.6-fold (*p* < 0.001) at 24, 48, and 72 h, respectively.

For SKOV3 cells in the scrambled control group, the wound closure rates at 24, 48, 72, and 96 h were 82.3%, 78.7%, 67.0%, and 54.2%, respectively (Figure 5A). In the siEGFL8#1 group, the closure rates were 78.5%, 66.4%, 62.3%, and 49.6%, while in the siEGFL8#2 group, they were 75.1%, 56.9%, 45.7%, and 27.0% (Figure 5A). These results indicate that the migration ability of the siEGFL8#1 group was significantly suppressed at 24, 48, 72, and 96 h, with reductions of 0.9-fold (*p* < 0.001), 0.8-fold (*p* < 0.001), 0.9-fold (*p* < 0.001), and 0.9-fold (*p* < 0.001), respectively, compared to the scrambled control group. In the siEGFL8#2 group, the migration ability was decreased by 0.9-fold (*p* < 0.001), 0.7-fold (*p* < 0.001), 0.7-fold (*p* < 0.001), and 0.5-fold (*p* < 0.001) at 24, 48, 72, and 96 h, respectively (Figure 4B).

Additionally, transwell invasion assays showed that EGFL8 silencing with siEGFL8 significantly increased the number of invasive cells compared to non-transfected cells (Figure 5B). The cell invasion ability of the siEGFL8#2 group was significantly enhanced compared to that of the scrambled control group at 24 h for A2780 cells, with a 2.7-fold (*p* < 0.001) increase, indicating that EGFL8 suppression accelerated the migration and invasion of OC cells, underscoring EGFL8’s role in human OC cell metastasis.

### 2.6. Knockdown of EGFL8 Reduces Human OC Cell Apoptosis

**Figure 6:**
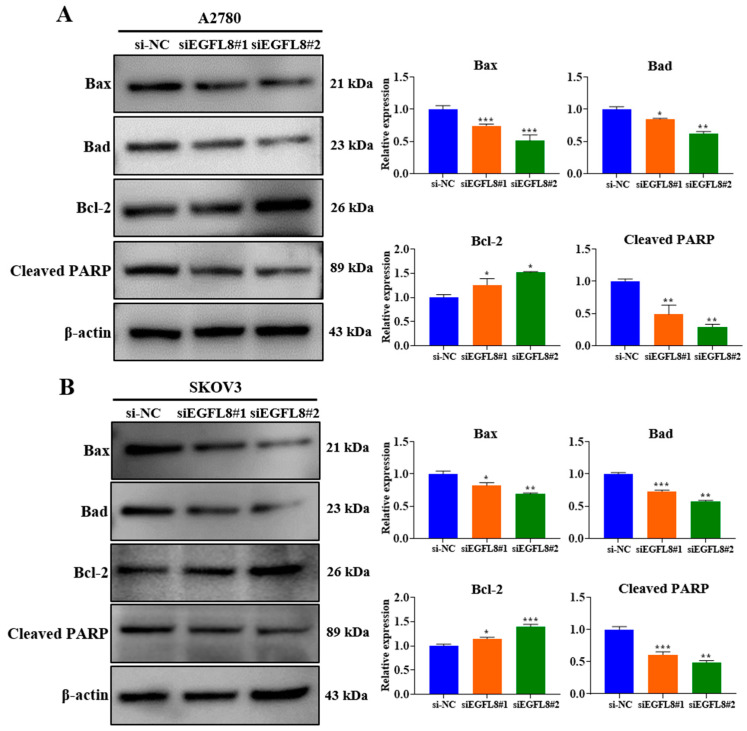
Effects of EGFL8 knockdown on apoptosis in human OC cells. Expression of apoptotic proteins in siEGFL8-transfected and non-transfected A2780 (**A**) and SKOV3 (**B**) cells was detected by Western blot analysis. Bar graphs depict densitometry quantitation of protein expression normalized to β-actin protein. Data represent mean ± SD of three independent experiments. * *p* < 0.05, ** *p* < 0.01, and *** *p* < 0.001 vs. respective scrambled control group.

Western blot analysis showed that EGFL8 downregulation in siEGFL8-transfected cells significantly induced apoptosis compared to that in the scrambled control group (Figure 6A,B). We identified that EGFL8 silencing markedly reduced levels of the pro-apoptotic proteins Bax and Bad in A2780 cells by 0.7-fold (*p* < 0.001) and 0.8-fold (*p* < 0.05), respectively, in the siEGFL8#1 group; and by 0.5-fold (*p* < 0.001) and 0.6-fold (*p* < 0.01), respectively, in the siEGFL8#2 group (Figure 6A,B). In SKOV3 cells, Bax and Bad were reduced by 0.8-fold (*p* < 0.05) and 0.7-fold (*p* < 0.001), respectively, in the siEGFL8#1 group; and by 0.6-fold (*p* < 0.01) and 0.5-fold (*p* < 0.01), respectively, in the siEGFL8#2 group (Figure 6A,B).

In contrast, EGFL8 silencing significantly increased the levels of the anti-apoptotic protein Bcl-2 in A2780 and SKOV3 cells by 1.2-fold (*p* < 0.05) and 1.2-fold (*p* < 0.05), respectively, in the siEGFL8#1 group; and by 1.3-fold (*p* < 0.05) and 1.4-fold (*p* < 0.001), respectively, in the siEGFL8#2 group (Figure 6A,B). As is consistent with these findings, EGFL8 silencing markedly suppressed cleaved PARP levels in A2780 and SKOV3 cells by 0.5-fold (*p* < 0.01) and 0.6-fold (*p* < 0.001), respectively, in the siEGFL8#1 group; and by 0.3-fold (*p* < 0.01) and 0.5-fold (*p* < 0.01), respectively, in the siEGFL8#2 group (Figure 6A,B). These results indicated that EGFL8 is critical in promoting apoptosis in human OC cells.

### 2.7. Knockdown of EGFL8 Augments Epithelial–Mesenchymal Transition (EMT) and Expression of Matrix Metallopeptidases

To explore the molecular mechanism by which EGFL8 regulates malignant cell behavior and tumor progression in human OC, quantitative Real-Time PCR (qRT-PCR) and confocal microscopy were performed to determine whether EGFL8 inhibits the expression of EMT-regulating transcription factors (TFs)—specifically, the pivotal EMT-TFs Snail, Twist1, and zinc-finger E homeobox-binding 1 (ZEB1); vimentin, a key biomarker of EMT; and matrix metallopeptidases (MMPs) such as MMP-2 and MMP-9.

**Figure 7:**
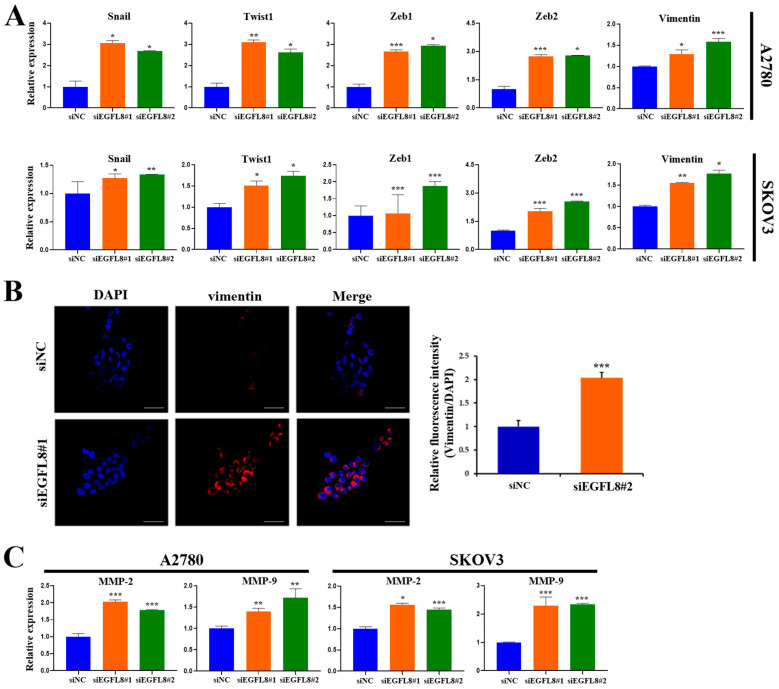
Effect of EGFL8 on expression of EMT-related markers and MMPs. (**A**) mRNA levels of key EMT markers in siEGFL8-transfected and non-transfected A2780 and SKOV3 cells as measured by qRT-PCR. (**B**) Confocal microscopy analysis of vimentin expressed in A2780 cells transfected with EGFL8 siRNA or scrambled siRNAs (blue: DAPI; red: vimentin). Scale bars = 30 μm. Bar graph shows quantification of red mean fluorescence intensity (MFI). Relative integrated mean fluorescence intensity (iMFI) of siEGFL8-transfected A2780 cells was normalized to that of scrambled negative control siEGFL8 (siNC). (**C**) Analysis of *MMP-2* and *MMP-9* expression in siEGFL8-transfected and non-transfected A2780 and SKOV3 cells by qRT-PCR. Bar graphs depict densitometric quantitation of mRNA expression normalized to *GAPDH*. Data represent mean ± standard deviation (SD) of three independent experiments. * *p* < 0.05, ** *p* < 0.01, and *** *p* < 0.001 vs. respective scrambled control group. EMT, epithelial–mesenchymal transition; MMP, matrix metallopeptidase.

We knocked down EGFL8 expression using siEGFL8#1. In A2780 cells, EGFL8 silencing significantly upregulated the expression of the key EMT transcription factors *Snail*, *Twist1*, *Zeb1*, and *Zeb2* by 3.1-fold (*p* < 0.05), 3.1-fold (*p* < 0.01), 2.7-fold (*p* < 0.001), and 2.7-fold (*p* < 0.001), respectively, compared to the scrambled control group (Figure 7A). Next, we knocked down EGFL8 using siEGFL8#2. In A2780 cells, EGFL8 silencing significantly upregulated the expression of the key EMT transcription factors *Snail*, *Twist1*, *Zeb1*, and *Zeb2* by 2.7-fold (*p* < 0.05), 2.6-fold (*p* < 0.05), 2.9-fold (*p* < 0.05), and 2.8-fold (*p* < 0.05), respectively, compared to the scrambled control group (Figure 7A). In SKOV3 cells, EGFL8 silencing with siEGFL8#1 significantly increased *Snail*, *Twist1*, *Zeb1*, and *Zeb2* expression by 1.3-fold (*p* < 0.05), 1.5-fold (*p* < 0.05), 1.1-fold (*p* < 0.001), and 2.0-fold (*p* < 0.001), respectively, compared to the scrambled control group (Figure 7A). EGFL8 silencing in SKOV3 cells using siEGFL8#2 significantly upregulated *Snail*, *Twist*, *Zeb1*, and *Zeb2* by 1.3-fold (*p* < 0.01), 1.7-fold (*p* < 0.05), 1.9-fold (*p* < 0.001), and 2.5-fold (*p* < 0.001), respectively, compared to the scrambled control group (Figure 7A).

Confocal microscopy further showed that EGFL8 downregulation in siEGFL8-transfected cells significantly enhanced vimentin expression compared to that in the scrambled control group (Figure 7B). EGFL8 silencing significantly increased vimentin expression by 2.1-fold (*p* < 0.001) in A2780 cells (Figure 7B). This finding was also confirmed by qRT-PCR, which showed 1.3-fold (*p* < 0.05) and 1.6-fold (*p* < 0.001) increases in *vimentin* expression in siEGFL8#1- and siEGFL8#2-transfected A2780 cells, respectively, and 1.6-fold (*p* < 0.01) and 1.8-fold (*p* < 0.05) increases in *vimentin* expression in siEGFL8#1- and siEGFL8#2 transfected SKOV3 cells, respectively (Figure 7A).

Furthermore, siEGFL8-transfected cells exhibited markedly increased levels of the key tissue remodeling proteases *MMP-2* and *MMP-9* by 2.0-fold (*p* < 0.001) and 1.4-fold (*p* < 0.01), respectively, in siEGFL8#1-transfected A2780 cells, and by 1.8-fold (*p* < 0.001) and 1.7-fold (*p* < 0.01), respectively, in siEGFL8#2-transfected A2780 cells. In SKOV3 cells, siEGFL8-transfected cells exhibited increased levels of *MMP-2* and *MMP-9* by 1.6-fold (*p* < 0.05) and 2.3-fold (*p* < 0.001), respectively, in siEGFL8#1-transfected cells, and by 1.4-fold (*p* < 0.001) and 2.3-fold (*p* < 0.001), respectively, in siEGFL8#2-transfected cells (Figure 7C). These findings suggested that EGFL8 is a crucial inhibitor of EMT and metastasis in human OC cells.

### 2.8. Knockdown of EGFL8 Upregulates Stem Cell Marker Expression

**Figure 8:**
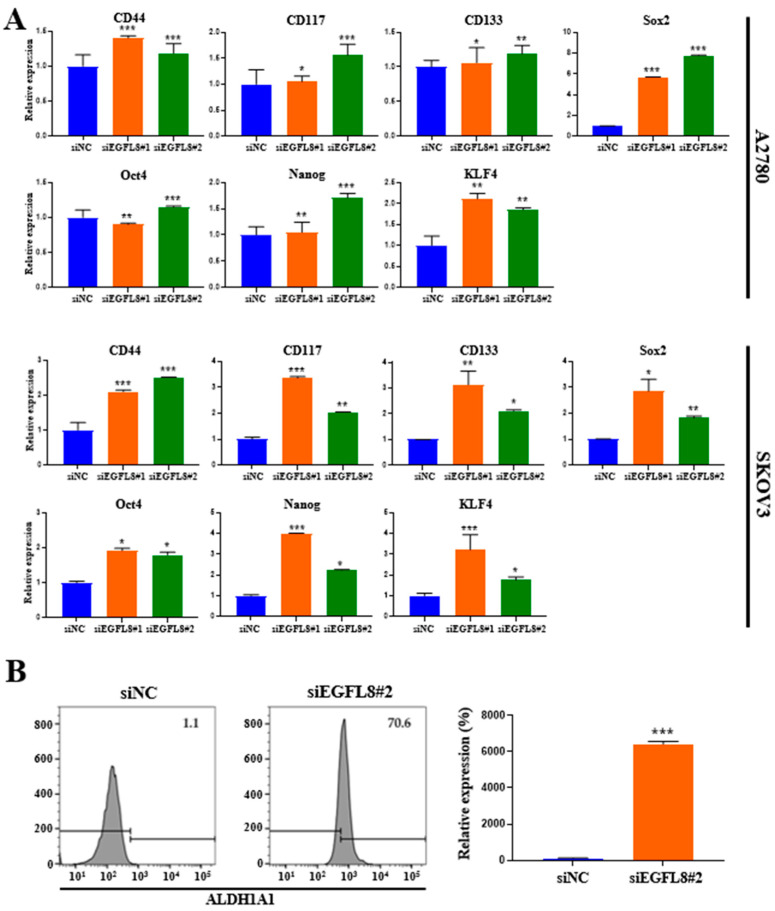
Effects of EGFL8 knockdown on expression of cancer stem cell (CSC) markers in human ovarian cancer (OC) cells. (**A**) Expression levels of *CD44*, *CD117*, *CD133*, *Sox2*, *Oct4*, *Nanog*, and *KLF4* in siEGFL8-transfected and non-transfected cells were analyzed by qRT-PCR. (**B**) Flow cytometry analysis of ALDH1A1 expression in siEGFL8-transfected and non-transfected A2780 cells. Data represent mean ± SD of three independent experiments. * *p* < 0.05, ** *p* < 0.01, and *** *p* < 0.001 vs. respective scrambled control group.

To elucidate the role of EGFL8 in cancer stemness, we analyzed the expression of genes associated with cancer stemness. Compared to the scrambled control cells, EGFL8 silencing via siRNA increased the expression of the common ovarian cancer stem cell (CSC) markers CD44, CD117, and CD133 and four major pluripotent stem cell transcription factors, sex-determining region Y-box 2 (SOX2), octamer-binding transcription factor-4 (OCT4), homeobox protein NANOG transcription factor (NANOG), and Krüppel-like factor 4 (KLF4) in human OC cells (Figure 8A). In A2780 cells, EGFL8 silencing significantly elevated mRNA levels of *CD44*, *CD117*, *CD133*, *Sox2*, *Oct4*, *Nanog*, and *KLF4* as measured by qRT-PCR, by 1.4-fold (*p* < 0.001), 1.1-fold (*p* < 0.05), 1.1-fold (*p* < 0.05), 5.7-fold (*p* < 0.001), 1.0-fold (*p* < 0.01), 1.1-fold (*p* < 0.01), and 2.1-fold (*p* < 0.01), respectively, in the siEGFL8#1 group; and by 1.2-fold (*p* < 0.001), 1.6-fold (*p* < 0.001), 1.2-fold (*p* < 0.01), 7.8-fold (*p* < 0.001), 1.1-fold (*p* < 0.001), 1.7-fold (*p* < 0.001), and 1.9-fold (*p* < 0.01), respectively, in the siEGFL8#2 group (Figure 8A).

In SKOV3 cells, EGFL8 silencing significantly upregulated the mRNA levels of *CD44*, *CD117*, *CD133*, *Sox2*, *Oct4*, *Nanog*, and *KLF4* by 2.1-fold (*p* < 0.001), 3.4-fold (*p* < 0.001), 3,1-fold (*p* < 0.01), 2.9-fold (*p* < 0.05), 1.9-fold (*p* < 0.05), 3,9-fold (*p* < 0.05), and 3.2-fold (*p* < 0.001), respectively, in the siEGFL8#1 group; and by 2.5-fold (*p* < 0.001), 2.0-fold (*p* < 0.01), 2.1-fold (*p* < 0.05), 1.8-fold (*p* < 0.01), 1.8-fold (*p* < 0.05), 2.2-fold (*p* < 0.05), and 1.8-fold (*p* < 0.05), respectively, in the siEGFL8#2 group (Figure 8A).

Furthermore, as is consistent with these findings, flow cytometric analysis revealed a strong increase in the level of aldehyde dehydrogenase 1 family member A1 (ALDH1A1), a functional stem cell marker, upon EGFL8 downregulation by 64.2-fold (*p* < 0.001) in A2780 cells (Figure 8B). These results suggested that EGFL8 plays a vital role in promoting human OC stem cell generation.

### 2.9. Knockdown of EGFL8 Enhances Chemoresistance

Overcoming multidrug resistance remains a critical challenge for effective cancer chemotherapy. Based on these results, we hypothesized that EGFL8 silencing would increase chemotherapeutic resistance in human OC cells. To test this hypothesis, siEGFL8-transfected and scrambled control A2780 and SKOV3 cells were cultured with serial concentrations of various human OC chemotherapeutic agents, including cisplatin, doxorubicin, docetaxel, paclitaxel, curcumin, and rucaparib, for 24 h. Cellular cytotoxicity, measured using a WST-1-based colorimetric viability assay, revealed that EGFL8 silencing significantly enhanced chemoresistance to these drugs.

**Figure 9:**
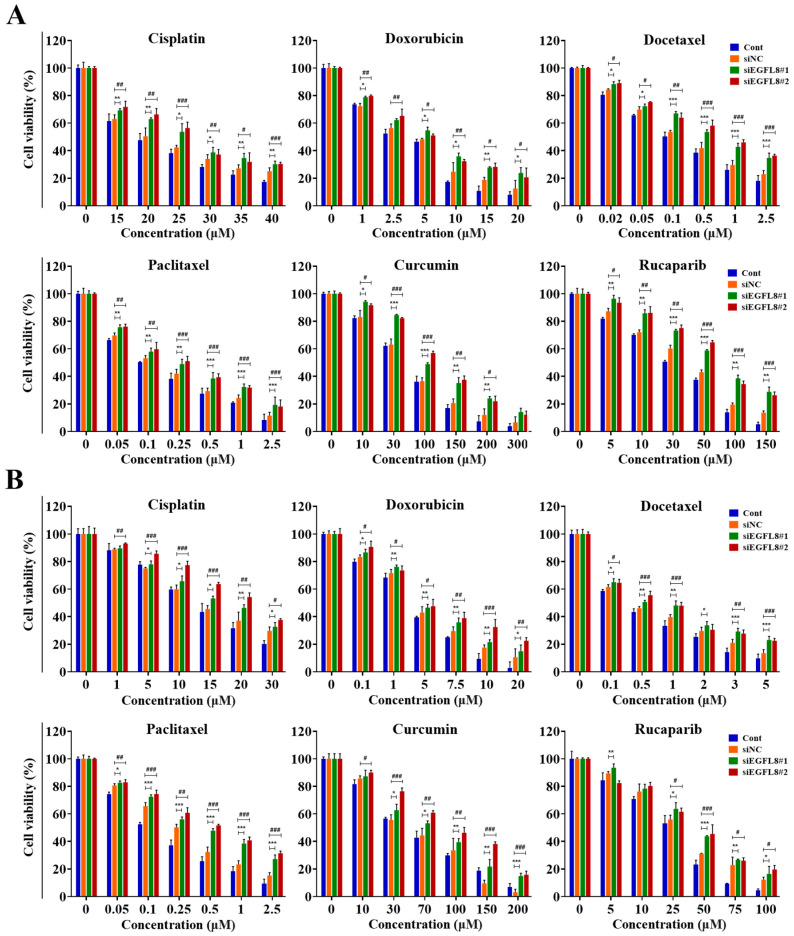
Effects of EGFL8 knockdown on chemosensitivity of cisplatin, doxorubicin, docetaxel, paclitaxel, curcumin, and rucaparib in A2780 (**A**) and SKOV3 (**B**) cells. Cell viability in scrambled control and siEGFL8-transfected human OC cells 24 h after drug treatment, as shown by WST-1 assay. Data represent mean ± SD of three independent experiments. * *p* < 0.05, ** *p* < 0.01, and *** *p* < 0.001, siEGFL8#1 vs. respective scrambled control group. ^#^ *p* < 0.05, ^##^ *p* < 0.01, and ^###^ *p* < 0.001, siEGFL8#2 vs. respective scrambled control group.

As shown in Figure 9, treatment with cisplatin, doxorubicin, docetaxel, paclitaxel, curcumin, or rucaparib for 24 h inhibited the viability of A2780 and SKOV3 cells in a dose-dependent manner. Cisplatin treatment (15–40 µM) in A2780 cells resulted in 1.1- to 1.3-fold (*p* < 0.01–0.05) greater cytotoxicity in the siEGFL8#1 group compared to the scrambled control group (Figure 9A). Similarly, 1.1- to 1.3-fold (*p* < 0.001–0.05) increases in cytotoxicity were observed in the siEGFL8#2 group compared to the scrambled control group (Figure 9A).

Doxorubicin treatment (1–20 µM) in A2780 cells resulted in 1.1- to 1.9-fold (*p* < 0.01–0.05) greater cytotoxicity in the siEGFL8#1 group compared to the scrambled control group (Figure 9A). Similarly, 1.1- to 1.7-fold (*p* < 0.01–0.05) increases in cytotoxicity were observed in the siEGFL8#2 group compared to the scrambled control group (Figure 9A).

Docetaxel treatment (0.02–2.5 µM) in A2780 cells resulted in 1.1- to 1.5-fold (*p* < 0.001–0.05) greater cytotoxicity in the siEGFL8#1 group compared to the scrambled control group (Figure 9A). Similarly, 1.1- to 1.6-fold (*p* < 0.001–0.05) increases in cytotoxicity were observed in the siEGFL8#2 group compared to the scrambled control group (Figure 9A).

Paclitaxel treatment (0.05–2.5 µM) in A2780 cells resulted in 1.1- to 1.7-fold (*p* < 0.001–0.01) greater cytotoxicity in the siEGFL8#1 group compared to the scrambled control group (Figure 9A). Similarly, 1.1- to 1.6-fold (*p* < 0.001–0.01) increases in cytotoxicity were observed in the siEGFL8#2 group compared to the scrambled control group (Figure 9A).

Curcumin treatment (10–300 µM) in A2780 cells resulted in 1.1- to 2.1-fold (*p* < 0.001–0.05) greater cytotoxicity in the siEGFL8#1 group compared to the scrambled control group (Figure 9A). Similarly, 1.1- to 1.9-fold (*p* < 0.001–0.05) fold increases in cytotoxicity were observed in the siEGFL8#2 group compared to the scrambled control group (Figure 9A).

Rucaparib treatment (5–150 µM) in A2780 cells resulted in 1.1- to 2.1-fold (*p* < 0.001–0.01) greater cytotoxicity in the siEGFL8#1 group compared to the scrambled control group (Figure 9A). Similarly, 1.1- to 1.9-fold (*p* < 0.001–0.05) increases in cytotoxicity were observed in the siEGFL8#2 group compared to the scrambled control group (Figure 9A).

Furthermore, cisplatin treatment (1–30 µM) in SKOV3 cells resulted in 1.0- to 1.2-fold (*p* < 0.01–0.05) greater cytotoxicity in the siEGFL8#1 group compared to the scrambled control group (Figure 9B). Similarly, 1.1- to 1.5-fold (*p* < 0.001–0.05) increases in cytotoxicity were observed in the siEGFL8#2 group compared to the scrambled control group (Figure 9B).

Doxorubicin treatment (0.1–20 µM) in SKOV3 cells resulted in 1.1- to 1.4-fold (*p* < 0.01–0.05) greater cytotoxicity in the siEGFL8#1 group compared to the scrambled control group (Figure 9B). Similarly, 1.1- to 2.1-fold (*p* < 0.001–0.05) increases in cytotoxicity were observed in the siEGFL8#2 group compared to the scrambled control group (Figure 9B).

Docetaxel treatment (0.1–5 µM) in SKOV3 cells resulted in 1.1- to 1.7-fold (*p* < 0.001–0.05) greater cytotoxicity in the siEGFL8#1 group compared to the scrambled control group (Figure 9B). Similarly, 1.1- to 1.7-fold (*p* < 0.001–0.05) increases in cytotoxicity were observed in the siEGFL8#2 group compared to the scrambled control group (Figure 9B).

Paclitaxel treatment (0.05–2.5 µM) in SKOV3 cells resulted in 1.1- to 1.8-fold (*p* < 0.001–0.05) greater cytotoxicity in the siEGFL8#1 group compared to the scrambled control group (Figure 9B). Similarly, 1.1- to 2.1-fold (*p* < 0.001–0.01) increases in cytotoxicity were observed in the siEGFL8#2 group compared to the scrambled control group (Figure 9B).

Curcumin treatment (10–200 µM) in SKOV3 cells resulted in 1.0- to 4.7-fold (*p* < 0.001–0.05) greater cytotoxicity in the siEGFL8#1 group compared to the scrambled control group (Figure 9B). Similarly, 1.1- to 5.0-fold (*p* < 0.001–0.05) increases in cytotoxicity were observed in the siEGFL8#2 group compared to the scrambled control group (Figure 9B).

Rucaparib treatment (5–100 µM) in SKOV3 cells resulted in 1.0-to 1.4-fold (*p* < 0.001–0.05) greater cytotoxicity in the siEGFL8#1 group compared to the scrambled control group (Figure 9B). Similarly, 0.9- to 1.6-fold (*p* < 0.001–0.05) increases in cytotoxicity were observed in the siEGFL8#2 group compared to the scrambled control group (Figure 9B).

We measured the IC_50_ values of cisplatin, doxorubicin, docetaxel, paclitaxel, curcumin, and rucaparib in A2780 and SKOV3 cell groups after 24 h of treatment. The IC_50_ values for these chemotherapeutic agents in the scrambled control and siEGFL8-transfected human OC cells are presented in Table 1.

In A2780 cells, EGFL8 silencing significantly elevated the IC_50_ value of cisplatin, doxorubicin, docetaxel, paclitaxel, curcumin, and rucaparib by 1.2-fold (*p* < 0.01), 1.5-fold (*p* < 0.05), 1.6-fold (*p* < 0.01), 1.5-fold (*p* < 0.001), 1.4-fold (*p* < 0.001), and 1.5-fold (*p* < 0.001), respectively, in the siEGFL8#1 group; and by 1.2-fold (*p* < 0.01), 1.5-fold (*p* < 0.05), 1.7-fold (*p* < 0.001), 1.5-fold (*p* < 0.001), 1.4-fold (*p* < 0.001), and 1.5-fold (*p* < 0.001), respectively, in the siEGFL8#2 group compared to the scrambled control group (Table 1).

In SKOV3 cells, EGFL8 silencing significantly increased the IC_50_ value of cisplatin, doxorubicin, docetaxel, paclitaxel, curcumin, and rucaparib by 1.2-fold (*p* < 0.01), 1.2-fold (*p* < 0.05), 3.1-fold (*p* < 0.001), 2.2-fold (*p* < 0.05), 1.3-fold (*p* < 0.01), and 1.2-fold (*p* < 0.01), respectively, in the siEGFL8#1 group; and by 1.4-fold (*p* < 0.001), 1.4-fold (*p* < 0.05), 3.3-fold (*p* < 0.001), 2.6-fold (*p* < 0.01), 1.6-fold (*p* < 0.01), and 1.2-fold (*p* < 0.05), respectively, in the siEGFL8#2 group compared to the scrambled control group (Table 1).

These results indicated that EGFL8 knockdown promotes resistance to multiple chemotherapeutic agents in human OC cells, further emphasizing EGFL8’s role in modulating drug sensitivity.

**Figure 10:**
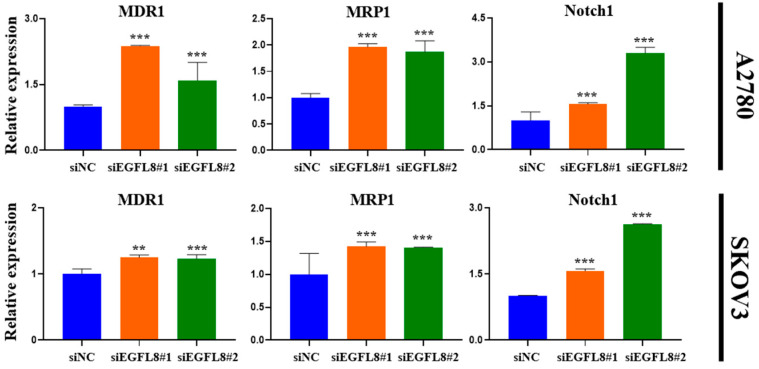
Effects of EGFL8 knockdown on expression levels of chemoresistance-related genes *MDR1*, *MRP1*, and *Notch1* in scrambled control and EGFL8 siEGFL8-transfected cells as measured by qRT-PCR. Bar graphs show densitometry quantification of mRNA expression normalized to *GAPDH*. Data represent mean ± SD of three independent experiments. ** *p* < 0.01 and *** *p* < 0.001 vs. respective scrambled control group.

Furthermore, gene expression analysis revealed a significant increase in the expression of multidrug resistance 1 (MDR1), multidrug resistance-associated protein 1 (MRP1), and Notch1 in EGFL8-silenced cells. In A2780 cells, EGFL8 silencing for 24 h significantly increased the expression of *MDR1*, *MRP1*, and *Notch1* by 2.4-fold (*p* < 0.001), 2.0-fold (*p* < 0.001), and 1.6-fold (*p* < 0.001), respectively, in the siEGFL8#1 group; and by 1.6-fold (*p* < 0.001), 1.9-fold (*p* < 0.001), and 3.3-fold (*p* < 0.001), respectively, in the siEGFL8#2 group compared to the scrambled control group (Figure 10). Similarly, in SKOV3 cells, EGFL8 silencing for 24 h significantly increased the expression of *MDR1*, *MRP1*, and *Notch1* by 1.2-fold (*p* < 0.01), 1.5-fold (*p* < 0.001), and 1.1-fold (*p* < 0.001), respectively, in the siEGFL8#1 group; and by 1.2-fold (*p* < 0.001), 1.4-fold (*p* < 0.001), and 2.2-fold (*p* < 0.001), respectively, in the siEGFL8#2 group compared to the scrambled control group (Figure 10).

These findings indicated that EGFL8 plays an essential role in mediating the resistance of human OC cells to various chemotherapeutic agents, including cisplatin, doxorubicin, docetaxel, paclitaxel, curcumin, and rucaparib.

### 2.10. Knockdown of EGFL8 Promotes Malignancy and Proliferation of Human OC Cells Through Activation of ERK/MAPK Signaling Pathway

To investigate the signal transduction pathway of EGFL8 in human OC cells, we tested various signaling cascades and found that EGFL8 activates the ERK/MAPK signaling pathway. We examined ERK1/2 phosphorylation in A2780 and SKOV3 cells following siEGFL8 transfection using Western blot analysis, both with and without U0126 (10 nM), a potent and selective inhibitor of MEK1/2 and ERK1/2 commonly used to inhibit the ERK/MAPK signaling pathway.

**Figure 11:**
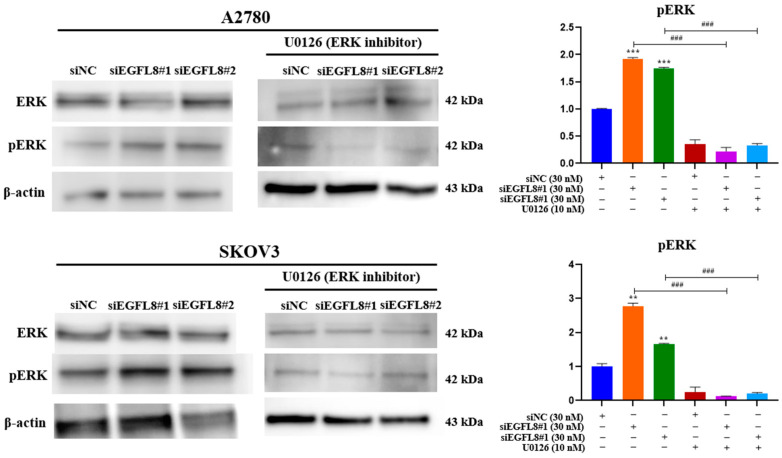
Effects of EGFL8 knockdown on ERK/MAPK pathway in human OC cells. Western blot analysis of ERK1/2 and phospho-ERK1/2 (pERK1/2) protein expression in scrambled control and siEGFL8-transfected A2780 and SKOV3 cells. Bar graphs display densitometric quantification of protein expression normalized to β-actin. Data presented as mean ± SD of three independent experiments. ** *p* < 0.01 and *** *p* < 0.001 compared to respective scrambled control group. ^###^ *p* < 0.001 compared to U0126-untreated siEGFL8-transfected A2780 and SKOV3 cells.

EGFL8 silencing by siEGFL8#1 and siEGFL8#2 transfection significantly increased ERK1/2 phosphorylation by 1.7-fold (*p* < 0.001) and 1.9-fold (*p* < 0.001) in A2780 cells, and by 2.2-fold (*p* < 0.001) and 1.7-fold (*p* < 0.001) in SKOV3 cells compared to that in the scrambled control group (Figure 11). Treatment with U0126 (10 nM) completely blocked siEGFL8-induced ERK activation, suggesting that EGFL8 knockdown activates the ERK/MAPK pathway in A2780 and SKOV3 cells (Figure 11).

**Figure 12:**
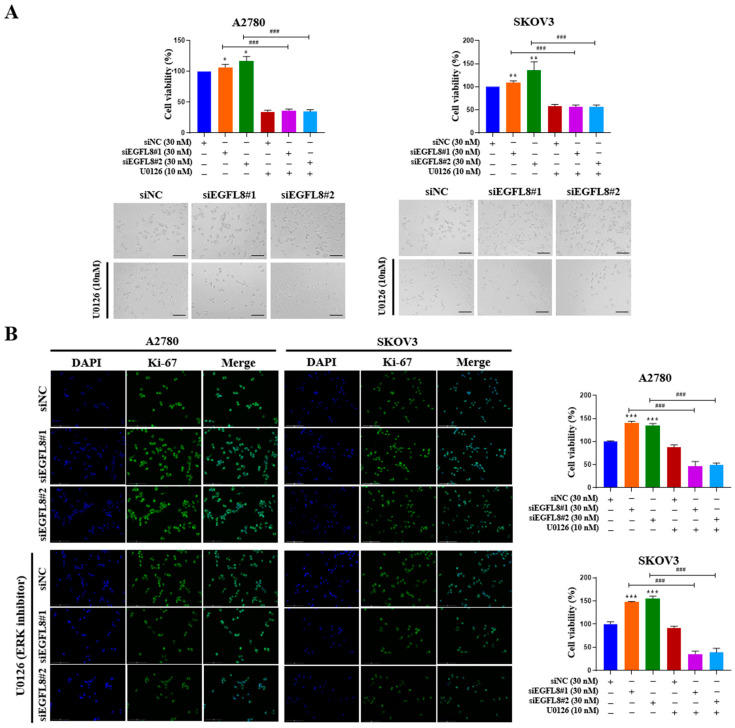
EGFL8 knockdown promotes human ovarian cancer (OC) cell proliferation by activating ERK/MAPK signaling pathway. (**A**) Results of WST-1 assay showing proliferation rates and representative phase-contrast microscopic images depicting cell morphology in scramble- and siEGFL8-transfected A2780 and SKOV3 cells at 24 h post-transfection. Scale bars = 150 μm. (**B**) Representative fluorescence micrographs of Ki-67 staining (blue: DAPI; green: Ki-67) in scramble- and siEGFL8-transfected A2780 and SKOV3 cells at 24 h. Scale bars = 150 μm. All data expressed as relative values compared to scrambled control group. Data represent mean ± SD of three independent experiments. * *p* < 0.05, ** *p* < 0.01, and *** *p* < 0.001 vs. respective scrambled control group. ^###^ *p* < 0.001 compared to U0126-untreated siEGFL8-transfected A2780 and SKOV3 cells.

To examine whether the siEGFL8-induced proliferation of A2780 and SKOV3 cells was regulated by the ERK/MAPK signaling pathway, the cells were transfected with siEGFL8#1 and siEGFL8#2 for 24 h in the presence or absence of U0126. The WST-1 cell proliferation assay was conducted to assess the effect of EGFL8 knockdown on cell proliferation. In both A2780 and SKOV3 cells, EGFL8 silencing for 24 h significantly increased cell proliferation compared to the scrambled control group (Figure 12A). Notably, this EGFL8 knockdown-induced cell proliferation was completely inhibited by U0126 (10 nM), a selective ERK/MAPK signaling blocker (Figure 12A). Additionally, these results were corroborated by morphological characteristics observed through phase-contrast microscopy (Figure 12A) and immunocytochemical staining for the cell cycle-associated nuclear antigen Ki-67 (Figure 12B). Confocal microscopy revealed that Ki-67 expression in A2780 cells following EGFL8 silencing for 24 h increased by 1.4-fold (*p* < 0.001) in both the siEGFL8#1 and siEGFL8#2 groups. The siEGFL8-induced increase in Ki-67 expression was effectively abrogated by treatment with U0126 (10 nM) (Figure 12B). These findings indicate that EGFL8 knockdown promotes cell proliferation via the ERK/MAPK signaling pathway in A2780 and SKOV3 cells.

### 2.11. Knockdown of EGFL8 Increases Expression of Malignancy-Related Molecules in Human OC Cells via Activation of ERK/MAPK Signaling Pathway

To investigate whether the ERK/MAPK signaling pathway is involved in the EGFL8-mediated upregulation of malignancy-related molecules in human OC cells, including EMT-regulating transcription factors (Twist1, Zeb1, and Zeb2), metastasis-promoting molecules (MMP-2, MMP-9, and Notch1), and stemness-regulating molecules (Oct4, CD44, and CD117), we performed qRT-PCR analysis in siNC- and siEGFL8-transfected A2780 and SKOV3 cells.

**Figure 13:**
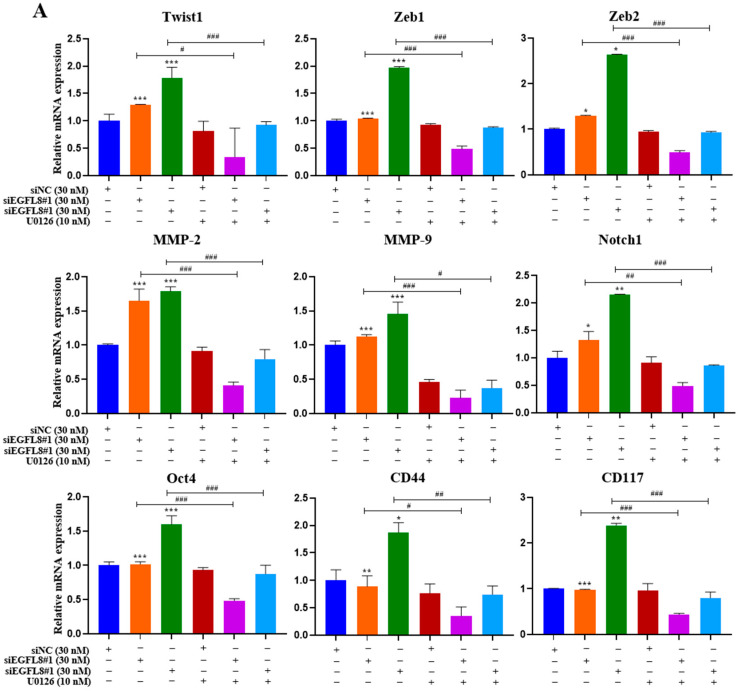

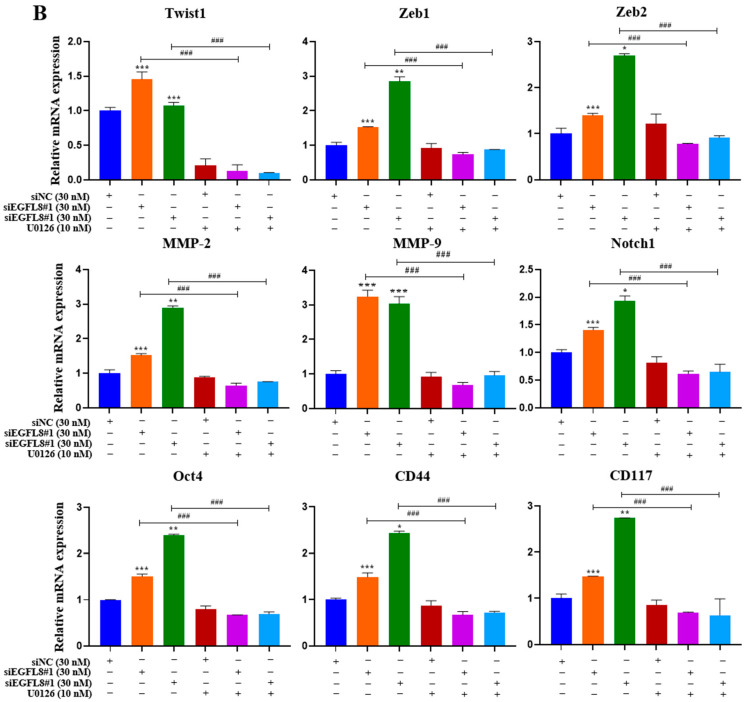
EGFL8 knockdown-mediated upregulation of EMT-regulating transcription factors, metastasis-promoting molecules, and stemness-regulating molecules in human OC cells acts via ERK/MAPK signaling pathway. Expression levels of key genes associated with malignancy in scramble- and siEGFL8-transfected A2780 (**A**) and SKOV3 (**B**) cells 24 h post-transfection measured using qRT-PCR. Bar graphs display relative mRNA expression levels of EMT-regulating transcription factors (*Twist1*, *Zeb1*, and *Zeb2*), (**B**) metastasis-promoting molecules (*MMP-2*, *MMP-9*, and *Notch1*), and stemness-regulating molecules (*Oct4*, *CD44*, and *CD117*) in A2780 and SKOV3 cells. *GAPDH* used as housekeeping gene for normalization. Data presented as relative values compared to respective scrambled control group and represent mean ± SD of three independent experiments. * *p* < 0.05, ** *p* < 0.01, and *** *p* < 0.001 vs. respective scrambled control group. ^#^ *p* < 0.05, ^##^ *p* < 0.01, and ^###^ *p* < 0.001 vs. U0126-untreated siEGFL8-transfected A2780 and SKOV3 cells.

Transfection with siEGFL8#1 and siEGFL8#2 for 24 h significantly upregulated the expression of all tested genes in both A2780 (Figure 13A) and SKOV3 (Figure 13B) cells. In both cell lines, the upregulation of *Twist1*, *Zeb1*, *Zeb2*, *MMP-2*, *MMP-9*, *Notch1*, *Oct4*, *CD44*, and *CD117* in the siEGFL8#1 and siEGFL8#2 groups was completely abrogated by 10 nM U0126 (Figure 13A,B).

## 3. Discussion

Previous studies have correlated chemoresistance, disease relapse, and frequent metastatic spread with high mortality and poor prognosis in patients with OC, one of the leading causes of cancer-related mortality in women [25,26]. Therefore, elucidating the molecular mechanisms and biological characteristics underlying human OC progression is critical for developing targeted therapeutics, identifying novel biomarkers, and improving patient prognosis.

This study is the first to investigate the biological function of EGFL8 in human OC cells, showing that EGFL8 is highly expressed in normal ovarian tissues, particularly in ovarian stromal cells as well as vascular endothelial and smooth muscle cells, but is significantly reduced in human OC tissues, with marked suppression of EGFL8 protein in stromal cells and minimal changes in vascular endothelial and smooth muscle cells. Despite the limited studies on EGFL8, as noted in the introduction, its characteristics and biological roles in normal and pathological cells remain largely unexplored. We previously discovered that the knockdown of EGFL8 in thymic epithelial cells enhanced thymocyte adherence by upregulating ICAM-1, promoting thymocyte maturation into CD4^+^ and CD8^+^ populations, and increasing the expression of thymopoiesis-related genes, including interleukin-7 (IL-7), granulocyte/macrophage-colony stimulating factor (GM-CSF), and thymus-expressing chemokine (TECK) [19]. These findings provide, for the first time, insight into EGFL8’s role as a negative regulator of T-cell development within the mouse thymus. Additionally, we revealed that EGFL8 negatively regulates thymocyte survival and proliferation by inducing apoptosis, inhibiting proliferation, and downregulating the Notch signaling pathway in thymocytes and thymic epithelial cells, demonstrating its functional role and molecular mechanism in these cells [20].

Furthermore, we found that EGFL8 knockdown in thymic epithelial cells upregulated several genes related to immune response, angiogenesis, and cell survival—such as CD74, Fas ligand (FasL), C-X-C motif chemokine ligand 5 (CXCL5), CXCL10, CXCL16, C-C motif chemokine ligand 20 (CCL20), vascular endothelial growth factor-A (VEGFL8-A), interferon regulatory factor 7 (Irf7), insulin-like growth factor binding protein-4 (IEGFL8BP-4), thrombospondin 1 (Thbs1) and nuclear factor κB subunit 2 (NF-κB2), CXCL5, CXCL10, VEGFL8-A, and anti-apoptotic molecules like Bcl-2 and Bcl-xL—and increased the expression of cell cycle regulators, including CDK1, CDK4, CDK6, and cyclin D1 [27]. These findings underscore the critical role of EGFL8 in regulating various physiological processes in thymocytes and thymic epithelial cells.

As is consistent with previous studies that observed an association between downregulated EGFL8 expression and malignant characteristics, including poor prognosis in gastric and colorectal cancers and hepatocellular carcinoma, our study also demonstrated that EGFL8 RNA expression levels correlate with overall survival (OS) and disease-specific survival (DSS) in human OC, as analyzed using patient samples from TCGA, GTEx, and the Human Protein Atlas data [21,22,23,28]. Moreover, Weiss et al. [24] demonstrated that stromal Schwann cells (SCs) in peripheral neuroblastic tumors such as ganglioneuromas and neuroblastomas share nerve repair-associated gene expression with repair SCs, indicating their inherent plasticity. Neuroblastoma cells exposed to repaired SCs showed increased differentiation and reduced proliferation, with EGFL8 identified as a key factor promoting neurogenesis [24]. These findings revealed that human SCs undergo adaptive responses during both nerve injury and tumor development, highlighting their shared regenerative potential.

Taken together, these results highlight the potential of EGFL8 as a biomarker for various cancers, including ovarian, gastric, colorectal, and hepatocellular carcinomas, as well as its biological functions in different cell types and tissues such as ovarian stromal cells, vascular endothelial cells, and smooth muscle cells. However, despite these findings, the role of EGFL8 in cancer remains largely unknown, particularly in human OC, where its specific function is yet to be elucidated. Further studies are needed to elucidate the characteristics and role of EGFL8 in ovarian stromal cells, vascular endothelial cells, and smooth muscle cells, which are beyond the scope of the present study.

Despite lower EGFL8 expression in human OC tissues, several human OC cell lines express EGFL8, suggesting a role in human OC pathogenesis. Among these cell lines, A2780 and SKOV3, commonly used human OC models [29], showed higher EGFL8 expression than the other lines tested and were thus chosen for further investigation in this study. We examined the biological effects of EGFL8 knockdown in these cells, and the results indicated that EGFL8 knockdown significantly enhanced malignant characteristics, including increased cell proliferation, spheroidogenesis, migration, invasion, colony formation, and chemoresistance; the increased expression of genes related to multidrug resistance, EMT, and cancer stemness; and elevated levels of molecules associated with tumor progression compared to the scrambled control group. Additionally, at the molecular level, EGFL8 silencing altered the expression of Bcl-2, Bax, Bad, and cleaved PARP, indicating that EGFL8 modulates human OC cell apoptosis by activating anti-apoptotic and suppressing pro-apoptotic proteins. Regarding spheroid formation, we previously demonstrated that human OC cell spheroids generated through 3D cell culture in hydrogels exhibit more malignant characteristics than those grown in 2D cultures. These include enhanced proliferation, migration, invasion, chemoresistance, reduced apoptosis, the upregulation of multidrug resistance genes, and the increased expression of EMT factors, Notch, and stemness markers [30]. Therefore, our findings that EGFL8 knockdown enhances the spheroid growth of human ovarian cancer cells suggest that EGFL8 plays a critical role in suppressing spheroidogenesis and, consequently, inhibiting the malignant progression of these cells. Collectively, these results demonstrate that EGFL8 negatively regulates human OC cell growth and malignant progression, highlighting EGFL8’s potential in developing new anti-cancer drugs and targeted therapies for human OC.

Resistance to cancer therapies is a major cause of treatment failure and relapse and is often driven by the rare but impactful presence of cancer stem cells (CSCs) [31]. CSCs are a small subset of cancer cells with self-renewing and proliferative capabilities; they initiate carcinogenesis, sustain tumor growth and heterogeneity, drive cancer progression, and contribute to malignant transformation, therapeutic resistance, metastasis, and recurrence [32,33,34,35]. Consequently, CSC-targeted therapies that disrupt the stemness and therapeutic resistance of CSCs are a vital focus of current cancer research aiming to improve treatment outcomes, prevent recurrence, and potentially achieve a cure by specifically eradicating CSCs [36,37]. Intriguingly, this study showed that EGFL8 knockdown upregulates the expression of key CSC markers, CD44, CD117, CD133, Sox2, Nanog, Oct4, KLF4, and ALDH1A1, highlighting EGFL8’s role in regulating CSC properties in human OC cells and suggesting its potential for developing targeted CSC therapies for human OC.

Metastasis is a complex, multi-step process involving the dysregulation of numerous genes and is the primary cause of cancer-related deaths, responsible for over 90% of fatalities, as cancer cells spread from the primary tumor to distant tissues [38]. This progression is often accompanied by resistance to conventional therapies, posing a significant challenge for effective treatment and underscoring the need for novel targeted therapies to combat both resistance and metastatic spread. To metastasize, tumor cells acquire invasive stem cell-like properties by hijacking the EMT program, gaining chemoresistance and stem-like traits regulated by EMT-inducing transcription factors such as Snail, Slug, Twist, and ZEB1/2 [38]. Cells with mesenchymal properties, characterized by actin and vimentin in their cytoskeleton, drive metastasis through enhanced migration and invasion [39]. EMT is a crucial step in the development of invasive and metastatic phenotypes in human cancers [40]. Given that EGFL8 knockdown promotes human OC cell migration and invasion, we further explored the molecular mechanisms underlying its role, particularly in EMT. We found that EGFL8 knockdown increased the expression of the critical EMT-regulating transcription factors Snail, Slug, Twist, ZEB1, ZEB2, and vimentin, suggesting that EGFL8 inhibits EMT in human OC cells.

Snail transcription factors play a key role in cancer progression, serve as primary EMT inducers, and are linked to a more aggressive phenotype and poor prognosis by influencing invasion, survival, proliferation, spheroid formation, stemness, chemoresistance, and metabolic changes in human OC cells [41,42]. Twist1 promotes ovarian cancer metastasis by upregulating discoidin domain receptor 2 (DDR2), a receptor tyrosine kinase (RTK) that recognizes fibrillar collagen as a ligand and enhances mesothelial clearance, invasion, and migration through matrix remodeling and Snail1 stabilization [43]. Twist1 also promotes stem-like cell differentiation by enhancing β-catenin degradation through Axin2 upregulation in human OC [44]. ZEB1 and ZEB2 are associated with tumor progression and malignancy in human OC by regulating EMT interconversions [45]. Vimentin is a key marker of EMT and plays a significant role in the aggressive phenotype of cancer cells, particularly in processes such as cancer progression, metastasis, and chemoresistance [46]. Additionally, MMP-2 and MMP-9, which facilitate the disassembly of collagen IV and laminin-5, which are key components of the epithelial basement membrane that regulate cell migration and invasion, were upregulated following EGFL8 silencing [47]. Both Snail and Slug contribute to EMT initiation by upregulating the expression of MMP-2 and MMP-9 [48]. Overall, these findings suggest that EGFL8 suppresses EMT and inhibits human OC cell metastasis.

Multidrug resistance, the primary cause of chemotherapy failure, limits drug efficacy and promotes tumor cell transformation into more aggressive or metastatic forms; it remains a major obstacle to cancer treatment and is a leading cause of cancer-related deaths [49,50]. Chemotherapy resistance involves several mechanisms, one of which is the enhanced efflux of drugs from cells [51]. Membrane transporter proteins such as MDR1 (P-glycoprotein, P-gp, or ATP-binding cassette subfamily B member 1, ABCB1) and MRP1 (ATP-binding cassette subfamily C member 1, ABCC1) play crucial roles in this process. These proteins actively export chemotherapeutic drugs from cells thereby diminishing their effectiveness and reducing cellular exposure to therapeutic agents [52]. The overexpression of multidrug-resistant proteins such as MDR1 and MRP1 in tumor cells mediates chemoresistance, although the mechanism is not yet fully understood [53,54,55]. In line with these findings, we observed increased chemoresistance to various anti-cancer agents in human OC cells, along with the upregulation of the major drug resistance genes, MDR1 and MRP1, following EGFL8 knockdown in A2780 and SKOV3 cells. This suggests that EGFL8 plays a key role in attenuating chemoresistance in human OC cells, highlighting its potential as a novel approach for EGFL8-mediated chemosensitization of human OC. In addition, in the present study, we discovered that silencing EGFL8 in human OC cells upregulated the expression of Notch1. The Notch1 signaling pathway plays a vital role in human OC proliferation, EMT, migration, invasion, apoptosis, stem cell maintenance, tumorigenesis, neoplastic transformation, EMT, chemoresistance, and metastasis [56,57,58,59,60,61,62,63,64,65]. These findings, along with our results, align with those of Wu et al. [23], who reported that the downregulation of EGFL8 regulates hepatocellular carcinoma cell migration, invasion, and apoptosis through the activation of the Notch signaling pathway.

Mitogen-activated protein kinase (MAPK) cascades are critical signaling pathways that regulate numerous cellular processes, including proliferation, differentiation, development, stress response, apoptosis, gene expression, and steroidogenesis [66]. Among these pathways, the classical RAS/RAF/MEK/ERK signaling cascade plays a central role, particularly through the ERK/MAPK pathway, which is involved in embryonic development and EMT to drive fibrosis and metastasis. In human OC, the ERK/MAPK pathway regulates essential cellular functions such as proliferation, differentiation, cell cycle progression, apoptosis, migration, invasion, angiogenesis, and metastasis [67,68]. Studies have shown elevated MAPK/ERK expression in human OC tissues compared to adjacent normal tissues [69], and similar increases were observed in various human cancers, including those of the colon, breast, and lungs [70,71,72,73]. Persistent ERK/MAPK activation can promote the transformation of normal cells into cancerous cells, whereas the inhibition of this pathway was shown to revert cancer cells to a non-transformed state in vitro and reduce tumor growth in vivo, indicating a strong link between heightened ERK/MAPK activity and cancer progression [74].

Excessive ERK/MAPK activation is associated with chemoresistance in human OC. This pathway mediates the phosphorylation of the pro-apoptotic protein BAD, contributing to platinum resistance by inhibiting apoptosis [75,76]. Blocking ERK/MAPK activity restores the chemosensitivity of human OC cells [77]. Additionally, human OC tissues often show low levels of miR-508/miR-18a, along with increased ERK/MAPK expression, whereas miR-508 mimics were found to suppress ERK/MAPK, inhibit EMT, and reduce malignant cancer progression [69,78].

Our findings suggested that EGFL8 regulates the ERK/MAPK pathway in human OC cells. The knockdown of EGFL8 using siEGFL8 significantly increased the expression of pERK and malignancy-related molecules in human OC cells, including EMT-regulating transcription factors (Twist1, Zeb1, and Zeb2), metastasis-promoting molecules (MMP-2, MMP-9, and Notch1), and stemness-associated markers (Oct4, CD44, and CD117). The upregulated expression of these genes and cell proliferation were completely abrogated by U0126, a specific MEK/ERK inhibitor, indicating that EGFL8 silencing specifically activated the ERK/MAPK pathway in both A2780 and SKOV3 cells. Therefore, EGFL8 negatively regulates human OC cell proliferation and cancer aggressiveness by inhibiting the ERK/MAPK signaling pathway.

In summary, this study is the first to show that EGFL8 expression is downregulated in human OC tissues compared to that in normal ovarian tissues, altering human OC cell characteristics and promoting metastatic properties via ERK/MAPK pathway activation. Our results highlight EGFL8 as a promising biomarker for predicting malignant characteristics, including cell proliferation, colony formation, migration, invasion, apoptosis, EMT, stemness, chemoresistance, metastasis, and prognosis, in human OC. Consequently, EGFL8 may serve as a valuable prognostic marker and a potential therapeutic target in human OC. However, further studies are necessary to elucidate the precise regulatory relationship between EGFL8 and the ERK/MAPK signaling pathways.

## 4. Materials and Methods

### 4.1. Cell Culture

The human OC cell lines CAOV3, SKOV3, and OVCAR3 were purchased from the American Type Culture Collection (ATCC, Manassas, VA, USA), and the A2780 cell line was obtained from the European Collection of Cell Cultures (ECACC, Salisbury, UK). R182, a chemotherapy-resistant human EOC cell line originating from malignant ovarian ascites, was a generous gift from Dr. J. Shah (Memorial Sloan-Kettering Cancer Center, New York, NY, USA). All cell lines were cultured and maintained in RPMI 1640 (Hyclone, Chicago, IL, USA), supplemented with 10% fetal bovine serum (FBS; Welgene, Daegu, Korea) and 1% penicillin-streptomycin (Thermo Fisher Scientific, Waltham, MA, USA) in a 5% CO_2_ humidified atmosphere at 37 °C. The medium was changed every third day.

### 4.2. Small Interfering RNA and Transfection

Human predesigned EGFL8 siRNA Set A, which contains three designed small interfering (si) RNA for EGFL8, as well as a scrambled siRNA (negative control), was purchased from MedChemExpress (Monmouth Junction, NJ, USA). Transfection procedures involving siRNA were performed according to the manufacturer’s protocol and as described [79], with some modifications. The number of A2780 cells was adjusted to 3 × 10^5^ cells/well. In 6-well plates, 2 mL of cell suspension was seeded and incubated overnight at 37 °C with 5% CO_2_. Subsequently, 30 nM siEGFL8 was added to 125 μL of a serum-free medium. Additionally, 9 μL of Lipofectamine™ RNAiMAX Transfection Reagent (Invitrogen, Waltham, MA, USA) was mixed with 116 μL of a serum-free media and incubated at 25 °C for 5 min. The solution containing siEGFL8s was then combined with the solution containing Lipofectamine™ RNAiMAX and gently mixed for 20 min at room temperature. The complex of siEGFL8 and Lipofectamine™ RNAiMAX was added to 6-well plates at 250 μL per well and mixed gently. After 24 h of transfection, the complex was removed, and the medium was replaced with a fresh medium.

### 4.3. Cell Proliferation Assay

A2780 and SKOV3 cells were seeded at a density of approximately 1 × 10^4^ cells/well in 96-well plates and cultured in complete medium containing 10% FBS for 24, 48, or 72 h. To measure cell proliferation, we performed a WST-1 colorimetric assay according to the manufacturer’s instructions (Daeil Lab Service, Seoul, Republic of Korea). In brief, the plates were washed with phosphate-buffered saline (PBS), 9 μL of WST-1 reagent was added to each well, and the plates were incubated for 1 h in a humidified chamber at 37 °C in 5% CO_2_. To quantify the metabolic cells, formazan absorbance was measured at 450 nm using a microplate reader (Tecan, Männedorf, Switzerland). Cell viability was calculated as the percentage of the control cell population (untransfected). Cell morphology was assessed at the desired time points using phase-contrast microscopy. All experiments were performed independently at least three times.

### 4.4. Colony-Forming Assay

Cells were seeded in 6-well plates at 600 cells/well and cultured for 7 days. Colonies were fixed with 100% methanol for 20 min at −20 °C and washed with PBS. The colonies were subsequently stained with 0.5% crystal violet solution (Sigma-Aldrich, St. Louis, MO, USA) for 5 min. The plates were washed twice with PBS and dried overnight. The stained colonies were counted to determine the number of colony-forming units (CFUs) using the EVOS M7000 imaging system (Thermo Fisher Scientific). For the colony counting assay, a colony was defined as a cluster consisting of at least 50 cells. Each experiment was independently repeated at least thrice.

### 4.5. Synthesis of Hydrogels for 3D Cell Culture

MC-B hydrogels were prepared as previously described to generate multicellular spheroids [30,80]. Briefly, 50 mg/mL sodium alginate was dissolved in deionized water for 16 h at room temperature (RT) to prepare a 5% alginate stock solution, which was autoclaved before use. Agarose (Affymetrix, Cleveland, OH, USA) was added to distilled water and heated to 100 °C to achieve a 2% agarose stock solution. Marine collagen (MC, Geltech, Busan, Republic of Korea) were added to distilled water at 0.3 g/mL, vortexed to dissolve, and resulted in a 30% marine collagen stock solution. A 565 μL volume of cells resuspended in culture medium (1 × 10^5^ cells/mL) was mixed with 240 μL of 5% sodium alginate solution in a 1.5 mL microcentrifuge tube at RT. This solution was then combined with 320 μL of 30% MC stock solution at RT to obtain 8% MC/1% alginate solution-containing cells. These cell suspensions were then blended carefully with 75 μL of 2% agarose solution at 35–40 °C to avoid cell damage. For the gelation of hydrogel solutions containing cells, the solutions were vortexed briefly, pipetted into 1 mL syringes, and ultimately incubated at 4 °C for 5–10 min. The gelled hydrogels were then transferred to the wells of 24-well plates (SPL Life Sciences, Pocheon, Republic of Korea) containing 1.5 mL of RPMI 1640 (Hyclone) and incubated at 37 °C. The medium was changed every 2 days.

### 4.6. Spheroid Growth Assay

To evaluate the effects of the MC-B hydrogels on the formation and growth of multicellular spheroids, A2780 and SKOV3 cells were cultured for 1, 4, 6, 10, 12, and 14 days, and 3, 5, and 7 days, respectively. Spheroid sizes were measured, and their morphology was photographed at the specified time points using a phase-contrast microscope (EVOS 7000, Thermo Fisher Scientific). Measurements were taken from at least 20 spheroids per hydrogel, and the average of the horizontal and vertical diameters was calculated to determine spheroid size.

### 4.7. Wound Healing Assay

Cells were seeded in 6-well plates at a density of 8 × 10^5^ cells/well, and after reaching complete confluency, they were scraped using a scratcher (SPL Life Sciences, Pocheon, Republic of Korea). After creating wounds in each well, the wells were washed with PBS to remove cellular debris and avoid the re-attachment of displaced cells. Scratch closure was monitored and imaged at 0, 24, 48, 72, and 96 h using a phase-contrast microscope. Each experiment was independently repeated at least thrice.

### 4.8. Invasion Assay

Cell invasion assay was assessed using a 24-well transwell chamber (8.0 μm pore size, SPL Life Sciences). Cells (5 × 10^4^) were resuspended in a serum-free medium and pipetted into the upper hanging chamber, and complete medium supplemented with 10% FBS was added to the lower chamber. After 24 h, the membranes were fixed and stained with a 0.5% crystal violet solution (Sigma-Aldrich). The uninvaded cells remaining in the upper chambers were removed using a cotton swab. The number of invading cells attached to the lower surface of the membrane was counted in five random fields using phase-contrast microscopy. All experiments were performed at least thrice.

### 4.9. Extraction of RNA and Quantitative Real-Time PCR (qRT-PCR)

Total RNA was isolated using TRIzol reagent (Favorgen Biotech Corp., Pingtung, Taiwan) following the manufacturer’s recommendations. The RNA quantity and quality were assessed by measuring the absorbance at 260 and 280 nm using a Nanodrop 2000 spectrophotometer (Thermo Fisher Scientific). Samples exhibiting an absorbance ratio (260/280) greater than or equal to 1.9 were used. First-strand cDNA was synthesized by reverse transcription from 1 µg of total RNA in a 20 μL reaction mix containing 0.5 µg oligo(dT) 12–18 primers (Promega, Madison, WI, USA), 50 mM Tris-HCl (pH 8.3), 75 mM KCl, 3 mM MgCl_2_, 40 mM dithiothreitol, 0.5 mM deoxynucleotide triphosphate (dNTP; Promega), 10 U RNase inhibitor (Promega), and 200 U Moloney murine leukemia virus (MMLV) reverse transcriptase (Promega). The reaction mixture was incubated at 37 °C for 60 min and then heated to 70 °C for 5 min to stop the reaction.

The CFX Connect Real-Time system (Bio-Rad, Hercules, CA, USA) and the DNA-binding dye SYBR Green I with SsoAdvanced Universal SYBR Green Supermix (Bio-Rad) were used for qRT-PCR. The primers used for qRT-PCR are listed in Table 2. All samples were amplified in triplicates. Gene expression levels were calculated using the 2^−ΔΔCt^ method and normalized to GAPDH expression levels. The expression of the control sample was set to 1, and the relative expression of the other samples was calculated accordingly.

### 4.10. Western Blot Analysis

To measure protein expression levels, the cells were harvested and washed twice with ice-cold PBS. The cells were then lysed in RIPA lysis buffer (GenDEPOT, Barker, TX, USA) containing a protease inhibitor cocktail (GenDEPOT) for 40 min on ice with agitation by vortex at 10 min intervals. The resulting homogenates were centrifuged at 13,000× *g* for 30 min at 4 °C, and the supernatants were collected. The protein concentration was determined using the bicinchoninic acid (BCA) protein assay (Sigma-Aldrich). An equal amount of protein per sample was mixed with Laemmli sample buffer (Bio-Rad, Hercules, CA, USA) and loaded (30 μg/lane) into a 10% (*v*/*v*) sodium dodecyl sulfate (SDS)-polyacrylamide gel electrophoresis (PAGE) gel. The separated proteins were blotted onto a polyvinylidene fluoride (PVDF) membrane (Amersham Biosciences, Piscataway, NJ, USA) using the semi-dry transfer method (Bio-Rad). The membranes were blocked with 3% bovine serum albumin (BSA) in Tris-buffered saline containing 0.1% Tween-20 at room temperature for 1 h. The membranes were washed and incubated overnight at 4 °C with primary antibodies against EGFL8 (AV42656, Sigma-Aldrich; Ab58650, Abcam, Cambridge, UK), Bax (Ab262929, Abcam, Cambridge, UK), Bcl-2 (Ab196495, Abcam), Bad (Ab90435, Abcam), Cleaved PARP (9541S, Abcam), ERK (4695S, Cell Signaling Technology, Danvers, MA, USA), pERK (4370S, Cell Signaling Technology), and β-actin (Sc-47778, Santa Cruz Biotechnology, Dallas, TX, USA) and subsequently incubated for 1 h at room temperature with anti-rabbit and anti-mouse secondary antibodies (7074S, 7076P2, Cell Signaling Technology). Following washing, the proteins were visualized using an enhanced chemiluminescence (ECL) kit (Dongin Biotech, Seoul, Republic of Korea) according to the manufacturer’s instructions. Images were captured using Amersham Imager 680 (Amersham Biosciences) and quantified using ImageJ software (version 1.52a, National Institute of Health, Bethesda, MD, USA).

### 4.11. Flow Cytometry

Fluorescence-activated cell sorting (FACS) was used to assess the effects of siEGFL8-mediated EGFL8 silencing on A2780 cells. Single-cell suspensions were prepared (1 × 10^5^) and washed with Hanks’ balanced salt solution (HBSS, Thermo Fisher Scientific) containing 0.1% BSA and 0.1% sodium azide. To detect the stem cell population, cells were stained with the phycoerythrin (PE)-conjugated rabbit cell surface marker ALDH1A1 monoclonal antibody (mAb; 65583S, Cell Signaling Technology) for 45 min at 4 °C in the dark with constant agitation on an orbital shaker at 200 rpm. The samples were then washed twice with HBSS containing 0.1% BSA and 0.1% sodium azide and resuspended. FACS was performed using a FACSCanto-II flow cytometer (BD Biosciences, San Jose, CA, USA). Flow cytometry data were analyzed using FlowJo 10.3.0 (Tree Star, Ashland, OR, USA).

### 4.12. Fluorescence Microscopy

For fluorescence microscopy analysis, cells cultured in an 8-well chamber (SPL Life Sciences) were fixed for 20 min with 4% paraformaldehyde. The fixative was removed by washing thrice with cold PBS (5 min each). The cells were permeabilized with 0.1% Triton X-100 in PBS for 5 min. After further washing with cold PBS, the cells were blocked with 2% BSA (Sigma-Aldrich) for 1 h at room temperature. The blocking solution was removed, and samples were incubated overnight at 4 °C with a rat Ki-67 mAb (14-5698-82, Thermo Fisher Scientific) and a mouse anti-vimentin mAb (sc-6260, Santa Cruz Biotechnology). After another wash with cold PBS, the cells were incubated for 1 h at room temperature with a rat secondary antibody (BioActs, Incheon, Republic of Korea) and a mouse secondary antibody (Thermo Fisher Scientific). After washing for a final time, the coverslips were mounted onto glass slides using Vectashield^®^ containing DAPI (Vector Laboratories, Burlingame, CA, USA) and imaged using an EVOS M7000 imaging system (Thermo Fisher Scientific). For quantification of the relative percentage of Ki-67^+^ cells, the number of Ki-67-stained cells was divided by the total number of DAPI-stained cells. Cells lacking green fluorescence (Ki-67 staining), exhibiting weak Ki-67 staining, or stained only with DAPI were excluded from the analysis. Similarly, for the quantification of vimentin staining, the integrated mean fluorescence intensity (iMFI) was calculated by multiplying the relative stained area of vimentin by the mean fluorescence intensity (MFI).

### 4.13. Chemotherapeutic Sensitivity Assay

After gene knockdown, the cells were seeded in 96-well plates at a density of 1 × 10^4^ cells/well. After allowing them to settle for 24 h, the cells were treated with 0, 15, 20, 25, 30, 35, and 40 μM cisplatin (Sigma-Aldrich); 0, 1, 2.5, 5, 10, 15, and 20 μM doxorubicin (Cell Signaling); 0, 0.02, 0.05, 0.1, 0.5, 1, and 2.5 μM docetaxel (Sigma-Aldrich); 0, 0.05, 0.1, 0.25, 0.5, 1, and 2.5 μM paclitaxel (Enzo Life Sciences, Farmingdale, NY, USA); 0, 10, 30, 100, 150, 200, and 300 μM curcumin (Sigma-Aldrich); and 0, 5, 10, 30, 50, 100, and 150 μM rucaparib (Selleckchem, Houston, TX, USA) in a serum-free media for 24 h. To determine cell viability, a WST-1 assay was performed as previously described [81]. All experiments were performed independently and repeated at least three times.

### 4.14. ERK Inhibitor Treatment

The cells were seeded at a density of 3 × 10^5^ cells/well in 6-well plates and cultured in a complete medium containing 10% FBS and 1% penicillin-streptomycin. After the cells were allowed to settle overnight, 10 nM U0126 (Merck, Seoul, Republic of Korea) was pretreated to a 2 mL a serum-free medium for 24 h before transfection.

### 4.15. Bioinformatics Analysis

The differential gene expression of EGFL8 between human OC and normal ovarian tissues was analyzed using the “Box Plot” feature within the “Expression Analysis” module of the Gene Expression Profiling Interactive Analysis (GEPIA2) platform (http://gepia2.cancer-pku.cn/, accessed on 7 October 2024) under default conditions, with a log_2_FC cutoff of 1, a *p*-value threshold of 0.01, and data from matched TCGA normal and GTEx data [82,83]. Protein expression levels of EGFL8 in human OC and normal ovarian tissues were retrieved from the Human Protein Atlas (HPA) version 24 (https://www.proteinatlas.org/, accessed on 10 October 2024), a comprehensive repository featuring immunohistochemistry (IHC)-based protein expression profiles across cancerous and normal tissues, as well as various cell lines [84,85]. IHC images from 18 tumor tissues and 2 normal tissues were downloaded from HPA and analyzed using ImageJ software (version 1.54 g; National Institutes of Health, Bethesda, MD, USA) to quantify the stained areas in these images. To assess the prognostic relevance of EGFL8 in human OC, analyses for overall survival (OS) and disease-specific survival (DSS) were conducted using the cBioPortal tool (http://www.cbioportal.org/), with TCGA data accessed on 2 October 2024, from provisional datasets for all analyses. A z-score cutoff for EGFL8 expression (RNAseq) was applied to create optimal separation in survival outcomes between the low expression (−2.5 ≤ z-score ≤ −0.5) and high expression (0.5 ≤ z-score ≤ 2.5) groups, yielding the most significant log-rank *p*-value [86]. Kaplan–Meier survival plots within cBioPortal were used for OS and DSS analyses, and statistical significance was determined using a log-rank test with a *p*-value < 0.05. All statistical analyses were conducted using cBioPortal analytical tools.

### 4.16. Statistical Analysis

All quantitative results were expressed as mean ± standard deviation of at least three independent experiments. Comparisons between two groups were analyzed using Student’s *t*-test. A value of * *p* < 0.05, ** *p* < 0.01, *** *p* < 0.001, ^#^ *p* < 0.05, ^##^ *p* < 0.01, or ^###^ *p* < 0.001, were considered statistically significant.

## 5. Conclusions

In conclusion, this study demonstrated that the siEGFL8-mediated knockdown of EGFL8 in human OC cells promoted aggressive traits linked to cancer progression, including increased proliferation, colony formation, migration, invasion, chemoresistance, and reduced apoptosis. The knockdown also upregulated EMT- and CSC-related biomarkers and increased the expression of MMPs, drug-resistance genes, and Notch1. Additionally, low EGFL8 expression correlated with poor prognosis in human OC. To our knowledge, this is the first study to show that EGFL8 suppresses human OC proliferation and cancer aggressiveness by inhibiting ERK/MAPK signaling. Therefore, our findings suggest that EGFL8 could serve as a valuable prognostic biomarker and a potential target for developing novel human OC therapies.

## Figures and Tables

**Table 1:** IC_50_ for chemotherapeutic agents in scrambled control and siEGFL8-transfected human OC cells.

Compounds	A2780			SKOV3		
	si-NC	siEGFL8#1	siEGFL8#2	si-NC	siEGFL8#1	siEGFL8#2
Cisplatin	22.99	26.7	26.95	16.63	19.14	22.98
Doxorubicin	5.22	7.9	7.63	5.81	6.88	7.99
Docetaxel	0.81	1.3	1.41	0.28	0.87	0.93
Paclitaxel	0.50	0.74	0.76	0.45	1.02	1.17
Curcumin	100.36	136.36	136.77	64.9	84.46	104.02
Rucaparib	56.58	86.82	85.01	41.9	49.64	48.98

**Table 2:** qRT-PCR primer names and their sequences.

Primer	Sequences
*SNAIL*	Forward: 5′-ACTGCAACAAGGAATACCTCAG-3′
	Reverse: 5′-GCACTGGTACTTCTTGACATCTG-3′
*Twist1*	Forward: 5′-GTCCGCAGTCTTACGAGGAG-3′
	Reverse: 5′-GCTTGAGGGTCTGAATCTTGCT-3′
*ZEB1*	Forward: 5′-TTACACCTTTGCATACAGAACCC-3′
	Reverse:5′-TTTACGATTACACCCAGACTGC-3′
*ZEB2*	Forward: 5′-GCGATGGTCATGCAGTCAG-3′
	Reverse: 5′-CAGGTGGCAGGTCATTTTCTT-3′
*Vimentin*	Forward: 5′-CAAAGCAGGAGTCCACTGAG-3′
	Reverse: 5′-TAAGGGCATCCACTTCACAG-3′
*MMP-2*	Forward: 5′-TGACGGTAAGGACGGACTC-3′
	Reverse: 5′-ATACTTCACACGGACCACTTG-3′
*MMP-9*	Forward: 5′-CAGAGATGCGTGGAGAGT-3′
	Reverse: 5′-TCTTCCGAGTAGTTTTGG-3′
*CD44*	Forward: 5′-CTGCCGCTTTGCAGGTGTA-3′
	Reverse: 5′-CATTGTGGGCAAGGTGCTATT-3′
*CD117*	Forward: 5′-CACCGAAGGAGGCACTTACACA-3′
	Reverse: 5′-TGCCATTCACGAGCCTGTCGTA-3′
*CD133*	Forward: 5′-CACTACCAAGGACAAGGCGTTC-3′
	Reverse: 5′-CAACGCCTCTTTGGTCTCCTTG-3′
*Sox2*	Forward: 5′-GCTACAGCATGATGCAGGACCA-3′
	Reverse: 5′-TCTGCGAGCTGGTCATGGAGTT-3′
*Oct4*	Forward: 5′-CTTGAATCCCGAATGGAAAGGG-3′
	Reverse: 5′-GTGTATATCCCAGGGTGATCCTC-3′
*NANOG*	Forward: 5′-CTCCAACATCCTGAACCTCAGC-3′
	Reverse: 5′-CGTCACACCATTGCTATTCTTCG-3′
*KLF4*	Forward: 5′-CATCTCAAGGCACACCTGCGAA-3′
	Reverse: 5′-TCGGTCGCATTTTTGGCACTGG-3′
*MDR1*	Forward: 5′-GCTGTCAAGGAAGCCAATGCCT-3′
	Reverse: 5′-TGCAATGGCGATCCTCTGCTTC-3′
*MRP1*	Reverse: 5′-CCGTGTACTCCAACGCTGACAT-3′
	Forward: 5′-ATGCTGTGCGTGACCAAGATCC-3′
*Notch1*	Reverse: 5′-GGTGAACTGCTCTGAGGAGATC-3′
	Forward: 5′-GGATTGCAGTCGTCCACGTTGA-3′
*GAPDH*	Reverse: 5′-GGAGAAGGCTGGGGCTCAT-3′
	Forward: 5′-TGATGGCATGGACTGTGGTC-3′

## Data Availability

Please contact the corresponding author with any data inquiries.

## Abbreviations

ABCB1	ATP-binding cassette subfamily B member 1
ALDH1A1	Aldehyde dehydrogenase 1 family member A1
BSA	Bovine serum albumine
CCL20	C-C motif chemokine ligand 20
CFUs	Colony-forming units
CSC	Cancer stem cell
CXCL	C-X-C motif chemokine ligand
DDR2	Discoidin domain receptor 2
DSS	Disease-specific survival
ECL	Enhanced chemiluminescence
EGFL8	Epidermal growth factor-like domain 8
EMT	Epithelial–mesenchymal transition
EGFL	Epidermal growth factor
FACS	Fluorescence-activated cell sorting
GEPIA2	Gene expression profiling interactive analysis
GM-CSF	Granulocyte/macrophage-colony stimulating factor
HBSS	Hanks’ balanced salt solution
HPA	Human Protein Atlas
IEGFL8BP-4	Insulin-like growth factor binding protein-4
IHC	Immunohistochemistry
IL-7	Interleukin-7
iMFI	integrated mean fluorescence intensity
Irf7	Interferon regulatory factor 7
KLF4	Krüppel-like factor 4
MC	Marine collagen
mAb	Monoclonal antibody
MC-B	Marine collagen-based
MFI	Mean fluorescence intensity
MDR1	Multidrug resistance 1
MMPs	Matrix metallopeptidases
MRP1	Multidrug resistance-associated protein 1
NANOG	Homeobox protein NANOG transcription factor
NF-κB2	Nuclear factor κB subunit 2
OC	Ovarian Cancer
OCT4	Octamer-binding transcription factor-4
OS	Overall survival
PAGE	Polyacrylamide gel electrophoressis
PBS	Phosphate-buffered saline
PE	Phycoerythrin
pERK1/2	Phospho-ERK1/2
P-gp	P-glycoprotein
PVDF	Polyvinylidene fluoride
qRT-PCR	Quantitative Real-Time PCR
RTK	Receptor tyrosine kinase
SCs	Schwann cells
si	Small interfering
Si-NC	Scrambled negative control siRNA
siRNA	Small interfering RNA
SOX2	Sex determining region Y-box 2
TECK	Thymus-expressed chemokine
TECs	Thymic epithelial cells
TFs	Transcription factors
Thbs1	Thrombospondin 1
TNM	Tumor–node–metastasis
VEGFL8-A	Vascular endothelial growth factor A
WST	Water-soluble tetrazolium
ZEB1	Zinc-finger E homeobox-binding
